# Standardized measurements for monitoring and comparing multiphoton microscope systems

**DOI:** 10.1038/s41596-024-01120-w

**Published:** 2025-03-17

**Authors:** Robert M. Lees, Isaac H. Bianco, Robert A. A. Campbell, Natalia Orlova, Darcy S. Peterka, Bruno Pichler, Spencer LaVere Smith, Dimitri Yatsenko, Che-Hang Yu, Adam M. Packer

**Affiliations:** 1 Science and Technology Facilities Council, Octopus imaging facility, Research Complex at Harwell, Harwell Campus, Oxfordshire, UK; 2 Department of Neuroscience, Physiology and Pharmacology, University College London, London, UK; 3 Sainsbury Wellcome Centre, University College London, London, UK; 4 Allen Institute, Seattle, WA, USA; 5 Mortimer B. Zuckerman Mind Brain Behavior Institute, Columbia University, New York, NY, USA; 6 Independent NeuroScience Services INSS Ltd, Lewes, UK; 7 Department of Electrical and Computer Engineering, University of California Santa Barbara, Santa Barbara, CA, USA; 8 DataJoint Inc., Houston, TX, USA; 9 Department of Physiology, Anatomy, and Genetics, University of Oxford, Oxford, UK

## Abstract

The goal of this protocol is to improve the characterization and performance standardization of multiphoton microscopy hardware across a large user base. We purposefully focus on hardware and only briefly touch on software and data analysis routines where relevant. Here we cover the measurement and quantification of laser power, pulse width optimization, field of view, resolution and photomultiplier tube performance. The intended audience is scientists with little expertise in optics who either build or use multiphoton microscopes in their laboratories. They can use our procedures to test whether their multiphoton microscope performs well and produces consistent data over the lifetime of their system. Individual procedures are designed to take 1–2 h to complete without the use of expensive equipment. The procedures listed here help standardize the microscopes and facilitate the reproducibility of data across setups.

## Introduction

Multiphoton excitation was originally described in the year 1932^1^, and the first scanning multiphoton microscope was demonstrated in the year 1990^2^. Since then, this technology has been driven by the use of multiphoton microscopy for imaging deep into scattering tissues. Many research laboratories now use either custom-built multiphoton microscopes or instruments from commercial manufacturers. However, there are very few accessible tools and procedures for quantifying a multiphoton microscope’s performance. This quantification is essential for maintaining imaging performance over time, comparing instruments and rigorously reporting experimental methods to enable reproducibility.

Protocols that quantify microscope system performance are necessary for several reasons. First, as developers, early adopters, builders, users and facility managers, we have realized that there is not always a consensus on which metrics are needed nor agreement on best practices when performing the relevant measurements. We want to share practices that we think provide the most crucial measurements both efficiently and accurately. Such measurements can provide valuable diagnostic information about system performance, especially when characterization is performed regularly. Second, we aim to further push the transition of multiphoton microscopy from frontier technology to a routine tool. Third, we hope this effort will contribute to more reliable comparisons of results within and across laboratories. Fourth and finally, we aspire to engage manufacturers to specify their microscopes’ performance in similarly quantitative ways and develop better tools for such characterization. Overall, we desire to push the field to improve data quality and rigor for the purposes of efficiency, open science and reproducibility.

### Overview of the procedures

**Fig. 1:**
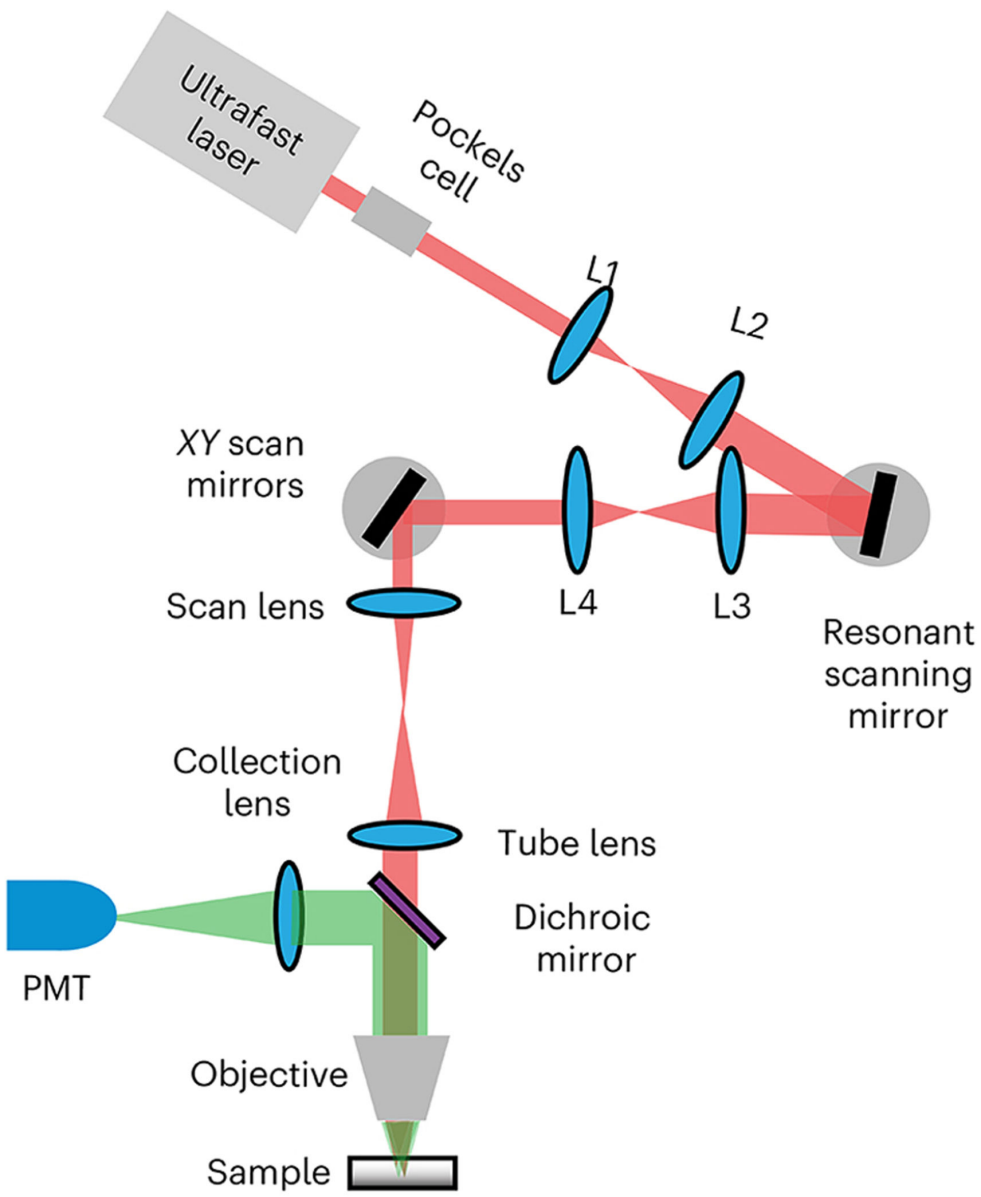
Basic schematic of a resonant-scanning multiphoton microscope. An ultrafast, pulsed laser (red) has its power modulated by the Pockels cell, after which the laser is relayed through the scanning system (lenses and mirrors) to the sample, through the objective. The sample undergoes fluorescence excitation and emission, and the emitted light (green) is separated from the original beam using a dichroic mirror to be directed at the detection system (in this case a photomultiplier tube; PMT).

Many of the procedures in this protocol can be performed independently, but some need to be performed before others in the first instance. Guidance on testing frequency is provided in the anticipated results section. We provide procedures to characterize both the excitation and collection subsystems of the microscope (Fig. 1). For excitation, we cover how to carefully and simply quantify the laser power on sample, the excitation volume (that is, point spread function (PSF)), the field of view (FOV) size and homogeneity and a protocol for optimizing excitation laser pulse width on sample. For collection, we provide a method for monitoring the sensitivity of photomultiplier tubes (PMTs), as well as one for quantifying the photon transfer function of the complete microscope system.

All procedures assume that the reader has all the components needed for a multiphoton microscope and that they are already configured correctly and controlled by software. While more detailed characterizations can be made with specialized (and expensive) equipment, we designed the procedures to require relatively simple, inexpensive and readily available resources.

### Laser power at the sample

Measuring and monitoring laser power at the sample is critical in multiphoton microscopy for maintaining sample integrity and data quality. First, emitted fluorescence, often the signal of interest, is proportional to the average laser power squared, so small changes in laser power can result in large changes to your data. Second, exposing your sample to excessive laser power can cause photobleaching and photodamage, altering your sample and measurements. Broadly, there are two types of laser-induced photodamage that can occur in multiphoton microscopy^3^. The first is the local heating of the area being imaged, which is linearly related to the average laser power. While this effect may be minor for normal imaging conditions, it should not be ignored, as it has been shown to alter the nature of biological samples^4–9^. The second type of photodamage is photochemical degradation, such as bleaching or even ablation, which is nonlinearly related to the average laser power^3,10,11^. Therefore, knowing the laser power is essential for consistency between experiments, for minimizing or eliminating photodamage and for monitoring the health of your imaging system.

Before reaching the microscope scan head, the laser beam is routed through a user-controlled variable power modulator (for example, a Pockels cell, motorized half-wave plate or acousto-optic modulator). The modulator may be a device that is integrated into the laser enclosure itself or a device that intercepts the laser beam path after it exits the enclosure. Attenuators typically use polarization or diffraction to send a defined proportion of the beam into a ‘dump’ to safely absorb excess light, while the remainder passes through the optical system and reaches the sample. When the user ‘changes laser power’ they are often altering the ratio of power going to the dump versus the sample.

Uncalibrated control software will usually allow the user to set laser power along an arbitrary scale, such as 0–100%. What those values mean exactly will depend on the software itself, but most likely, they simply map to the minimum and maximum of an analogue voltage output that is fed to the controller electronics of the power modulator. Some microscope control software allows the user to calibrate the on-screen laser power control such that it is mapped directly in watts, removing any nonlinearity introduced by the power modulation hardware. Other software has no facility for calibration and will always display power in arbitrary units. In this protocol, we describe the most generic process, which is to manually generate a table for converting percent power into a value in watts.

Microscopes can be configured and controlled in a variety of ways that can affect laser power measurements. There are often substantial losses in laser power as the beam traverses the path toward the sample. For example, it is not unusual for the objective alone to have transmission efficiencies of ~70% (refs. 12,13). For this reason, the laser power sensor is placed after the imaging objective to get the best estimate of power arriving at the sample. Note that laser power measurements will be dependent on the objective used and are only valid for that objective. A different objective will likely have different physical properties, such as antireflective coatings, different number of glass elements and a different back pupil diameter, all of which will affect transmission and, therefore, laser power at the sample.

There are two ways of measuring the time-averaged laser power under the objective: either with the beam stationary (typically centered in the imaging FOV) or with the beam continuously scanning. When possible, we recommend that measurements are made with a stationary beam or scanning with beam blanking disabled, as both conditions are independent of changes in effective duty cycle of the scanning (for example, from changes in zoom) and easily comparable across different microscopes and power meters. The effective average power on sample during an experiment (with beam blanking enabled) could also be measured and compared with values derived from the scan parameters and duty cycle (which should be provided in publications) to check they corroborate. We note that what is most critical is to use one method consistently and accurately report the experimental conditions under which the measurements were made.

### FOV size

In conventional laser scanning systems, the image is created by scanning the focused laser beam over a rectangular area with a set of mechanical scanners that steer the beam in the *X* (fast) and *Y* (slow) directions. This scan pattern is known as raster scanning. The beam travels over the rectangular area within the sample, exciting fluorophore molecules along the path of the scan. The resulting fast fluorescence emission (often a few nanoseconds) is detected and integrated over a short duration (the pixel dwell time) to create an intensity value for a single pixel. The consecutive pixels are captured and used to create an image.

The area on the sample covered by the scan is known as the FOV. The maximum area that can be scanned is limited by the optics within the microscope, the objective chosen and the scanning hardware, but under normal conditions is restricted by controlling the *X* and *Y* mirrors’ scan angle. This can be changed in software by changing the maximum galvanometer scan amplitude to ‘zoom in’ on some specific structure or ‘zoom out’ to see the maximum FOV. For additional information on the relation of FOV to the hardware parameters, see refs. 14–18. When using conventional galvanometers, the speed at which the scanners trace a line can be varied by the user, allowing changes in the rate at which lines are scanned. For resonant galvanometers, the period is fixed by the mechanical properties of the scanner, and only the scan amplitude can be changed.

Knowing the physical size of the FOV allows the pixel-pitch in microns to be calculated. From this, you can measure physical dimensions of features in the imaged sample as well as select the correct zoom factor to achieve spatial sampling that takes advantage of the full optical resolution of the system (known as Nyquist sampling), if required. Furthermore, measuring FOV size is an essential step for characterization of the imaging system: measurements in other procedures of this protocol (for example, Procedures 3 and 4) will rely on an accurately calibrated FOV. Additionally, an uncalibrated system may have nonsquare pixels and measuring the pixel size is a prerequisite for correcting this should it be necessary.

### FOV homogeneity

FOV homogeneity is a metric that defines the system’s uniformity of excitation and collection of light across the FOV of the sample. Measuring field homogeneity is easy to do and can be used to understand the data quality across the image and to know the experimental FOV size that minimizes this variation. Large, structured variations in FOV homogeneity can also highlight potential problems with microscope alignment or debris on optics in the path.

One common inhomogeneity that can be revealed from this measurement is known as ‘vignetting’, where signal is reduced at the edges of the FOV. Vignetting is important to consider when choosing the working FOV for experiments as reduced signal at the edges of the FOV will cause a decrease in signal-to-noise in those regions.

Vignetting is unavoidable when using large scan angles that are at the design limit of the microscope hardware and optics. The limits are defined by the optical properties of the microscope, particularly the objective lens. Manufacturers usually specify the FOV size over which an objective’s performance is said to be ‘diffraction limited’. Beyond the specified FOV size, the objective’s ability to focus light degrades and causes vignetting and other optical aberrations. Typically the objective is the principle limit for the FOV size, but an improperly designed excitation or collection path might become a limiting factor depending on PMT sensor size, angle of acceptance and the collecting lenses (for more on this topic, see refs. 19,20).

### Spatial resolution

Laser scanning imaging systems such as multiphoton microscopes provide spatial resolution governed by their PSF. The PSF is the impulse response of an imaging system. In other words, the PSF describes how a point object (an ‘impulse’) in the object space will be ‘spread’ by the imaging system and, thus, appear in the acquired image. The PSF is a three-dimensional spatial function whose shape can vary depending on the focusing optics of a system and across the FOV. Moreover, nonlinear excitation and excitation saturation can influence the effective spatial resolution in multiphoton microscopy^21^. Thus, in this protocol, the excitation volume geometry means the shape of the volume that is excited by the focused laser light at a given point in time. The geometry of the excitation volume governs how the smallest features will appear in the microscope and the effective spatial resolving power of the microscope. In multiphoton imaging the shape of the excitation volume is commonly characterized, often approximated by the full-width at half-maximum (FWHM) of the axial and radial profiles, and that is the procedure we describe in this protocol.

The measurement of an excitation volume matters because it indicates how an imaging system resolves signals from structures in the sample, and the profile of the excitation volume can be engineered depending on the requirement of the application. For common-use cases, the imaging system is designed to achieve a diffraction-limited PSF (the highest resolution possible for a system limited due to the physics of diffraction), which would ideally be smaller than the structures of interest being observed^16,21,22^. In this way, the emission from the excitation volume is dominated by signals from individual structures, and contributions from neighboring structures are minimal. We note that the term ‘diffraction limited’ is typically applied to beams with planar or gaussian wavefronts that then enter and are focused by standard objectives. In contrast, there are purposefully designed nondiffraction-limited PSFs, which match or are much larger than the structure of interest; these are utilized to rapidly sample volumes of tissue, especially when staining is bright and sparse^23–28^. Regardless of the experimental approach, excitation volume geometry should be characterized and monitored over time to ensure consistent resolution within a set of experiments. Although the example shown in this protocol is the diffraction-limited case, this protocol is empirical and applies in nondiffraction-limited cases as well.

The numerical aperture (NA) is a unitless parameter that characterizes the range of ray angles that an optical system can accept or emit. The NA and wavelength of the excitation light are the major factors that influence the smallest possible spatial extent of the PSF, such that larger NAs and shorter wavelengths lead to smaller PSFs. The maximum possible NA is set by the objective. However, if the excitation light entering the objective underfills the back pupil, then the effective excitation NA will be reduced, and the actual PSF is larger than the theoretical, diffraction-limited prediction for that objective^29,30^. The profile of the beam used to illuminate the back aperture is approximately Gaussian in most cases, and its width is commonly characterized by the spatial extent over which the intensity exceeds 1/e^2^ of its maximum (where ‘e’ is the base of the natural logarithm). Thus, to use the full NA of the objective, the back pupil of the objective must be overfilled, leading to some power being discarded. Sometimes objectives are deliberately underfilled to transmit more power or for application-specific PSF engineering strategies; other times, underfilling is unintentional and due to clipping or limiting apertures in the microscope optical path. In either case, the excitation volume will have a larger spatial extent than predicted by theory for the objective’s listed NA.

**Fig. 2:**
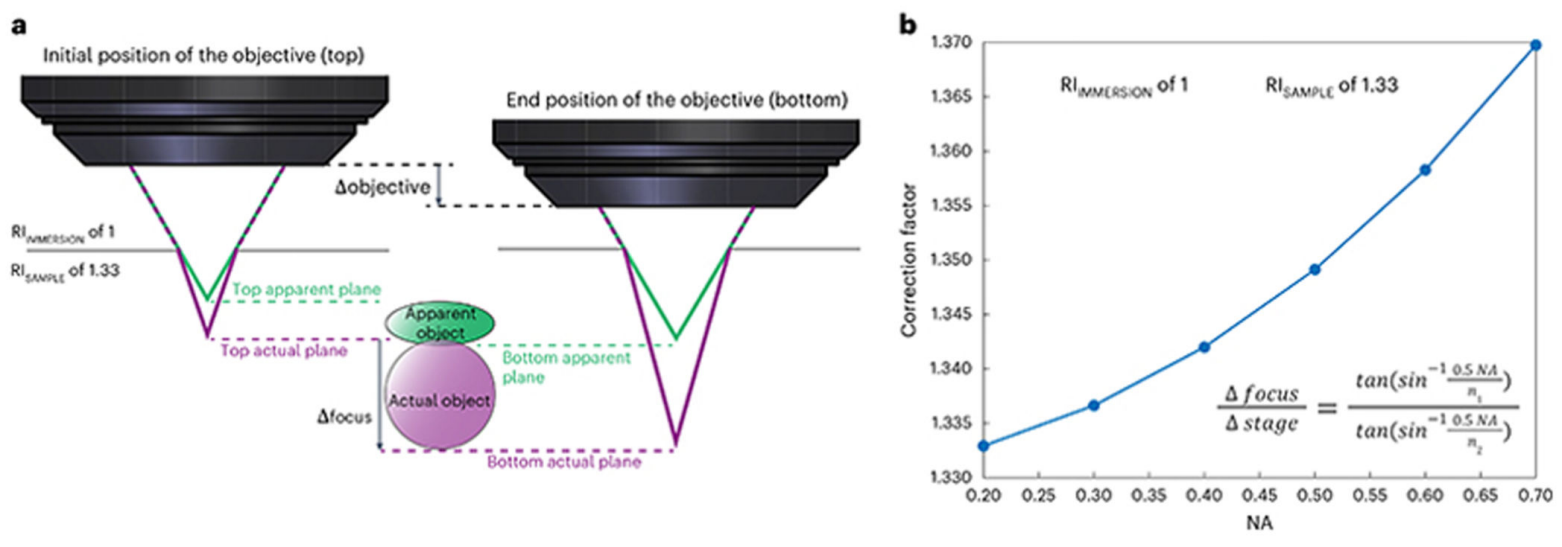
Object distortion caused by RI mismatching and the correction factor. **a**, Scanning the objective lens axially (indicated by the black arrow, Δobjective) through a spherical object (labeled as the ‘actual object’ in purple, Δfocus) submerged in water produces a three-dimensional image. Despite this, the image (labeled as the ‘apparent object’ in green) appears compressed along the axial direction. This compression arises due to the air-to-water interface, where the RI transitions from air (RI_IMMERSION_ of 1) to water (RI_SAMPLE_ of 1.33), causing light rays (purple rays) to bend. Consequently, the movement of the objective (Δobjective) is smaller than that of the actual focal plane (Δfocus). The resulting rendering of the sphere (‘apparent object’, green) exhibits axial compression, as the acquisition software assigns the objective’s travel distance (Δobjective) rather than the focal plane’s travel distance (Δfocus) to the object’s *z* axis. A correction factor can be calculated and applied to correct this distortion. **b**, A plot shows the correction factor converting the movement of the objective (Δobjective) or the *z*-stage (Δstage) to that of the actual imaging plane (Δfocus) as a function of NA of the objective. RI_IMMERSION_ = *n*_1_ = 1 and RI_SAMPLE_ = *n*_2_ = 1.33. The figure is adapted from ref. 34, Springer Nature Ltd.

Besides the NA and wavelength, other experimental and sample specific factors affect the resolution. For example, the refractive index (RI) varies for materials. Air has an RI close to 1, water has an RI ~1.33 and different types of glass can exhibit RIs in the range from ~1.5 to 1.9. When light rays are converging to a focus, discontinuities or changes in the RI can cause marginal rays to be refracted more strongly than rays close to the optical axis, thus degrading the quality of the focus. This condition is a type of optical aberration called spherical aberration (SA), and SA will reduce the actual spatial resolution from the theoretical prediction. This resolution degradation is typically more pronounced in the axial direction, the direction along which PSFs often have their longest spatial extent. SA can be reduced by matching the RIs of the immersion medium and the sample, when possible. Some microscope objectives correct for some SA through compensation mechanisms such as a correction collar, which can restore diffraction-limited imaging over a range of RI mismatches. In other systems, adaptive optics such as deformable mirrors can compensate for such aberrations^31,32^. Although SA could be corrected, RI mismatch also gives rise to a different focal shift for the imaging plane from the traveling distance of the stage (or the objective) along the *z* axis (Fig. 2). This unequal displacement results in axially distorted images, either compressed or elongated. A correction factor can be calculated to correct for this axial distortion and should be applied^33,34^.

Overall, off-axis aberrations intrinsic to the lenses (for example, coma and astigmatism) gradually increase and deteriorate the geometry of the excitation volume toward the periphery of the FOV. The aberrations are the result of the combination of relay optics, objectives, the arrangement of scanners (closely coupled or separately conjugated orthogonal scanners) and so on. Measuring the resolution across the FOV is recommended, especially for a large FOV (>1 mm) imaging system.

It is worth noting that tissue scattering deteriorates the resolution in a depth-dependent manner, as the marginal rays out of the objective travel a longer distance than an on-axis ray and accumulate larger scattering and attenuation. The difference in traveling distance can result in a smaller effective NA and impacts the effective excitation volume as the imaging plane goes deeper, especially for high NA objectives^35,36^. This resultant degradation in signal and resolution can be more severe than simply starting with a lower effective NA and is a reason why it is generally recommended to underfill objectives when imaging deep^37,38^.

Note that the excitation volume measured in this protocol is focused on characterizing the imaging system only. Imaging biological specimens will entail additional aberrations and scattering, which can be compensated for by adaptive optics^39^.

### Pulse width control and optimization

Nearly all multiphoton microscopy makes use of modelocked^40^ ultrafast lasers with pulse durations on the order of 100 femtoseconds (100 × 10^-15^ s). Due to the nonlinearity of multiphoton excitation, peak intensity matters more than average power for efficient excitation. The efficiency of multiphoton excitation using pulsed lasers versus CW (continuous wave) lasers, with the same time-averaged power, is given by

(1) $$g^{\left(n\right)}=\frac{g_{\text{p}}^{\left(n\right)}}{{\left(\tau f_{\text{R}}\right)}^{n-1}},$$

where *g*^(*n*)^ is the enhancement factor, *τ* is the pulse width, *f*_R_ is the repetition rate and *n* is number of photons in the absorption process^16^. The factor $g_{\text{p}}^{\left(n\right)}$ depends only on the pulse shape and is equal to one for a rectangular pulse and 0.59 for a hyperbolic secant envelope, which is close to the typical shape of the pulses delivered from mode-locked pulsed lasers^40^. For two-photon imaging, with a standard Ti–sapphire laser operating at 80 MHz with 150 femtosecond pulses, this enhancement over CW lasers is ~50,000 and is strongly dependent on *τ*, the pulse width.

The generation of short laser pulses requires finite bandwidth—the shorter the desired laser pulse, the broader the spectral content. This can be rigorously derived classically through Fourier transform relationships and for the hyperbolic secant envelope mentioned above, which leads to *τ*_p_ × Δ*v*_p_ ≥≥ 0.3148, with *τ*_p_ the FWHM of the laser pulse envelope and Δ*v*_p_ the FWHM of the frequency spectrum^40^. Converting frequency to wavelength means a 150 fs pulse centered at 920 nm needs at least a FWHM bandwidth of 6 nm, a 100 fs pulse 9 nm and a 50 fs pulse 18 nm. It is important to note that broad bandwidth alone does not guarantee a short pulse, however it is required to generate one. Where the pulse is the shortest it can possibly be, given the nominal bandwidth envelope, the pulses are known as transform-limited pulses.

Once a transform-limited pulse leaves the laser, the pulse envelope and spectral phase can often be altered through interactions with materials that introduce dispersion, such as optical glasses. A dispersive material is one that has a frequency (wavelength) dependent RI. If the RI increases with increasing frequencies (decreasing wavelength) the material is said to have positive dispersion, whereas if the RI decreases with increasing frequency, it is said to have negative dispersion. At the common wavelengths used in microscopy, most materials exhibit positive dispersion^41^. The RI can be related to the speed at which light travels through a medium; for positively dispersive media, the bluer portion of the pulse travels at a slower velocity than the redder portion, temporally broadening or ‘stretching’ the pulse and resulting in a ‘chirped’ pulse. If there is a material with negative dispersion, the opposite occurs, and the redder part of the pulse is delayed relative to the bluer. For a given material, the more material that the pulse propagates through, the greater the temporal broadening or stretching. Microscopes with elaborate optical systems (such as large FOV systems^42^) have more or thicker glass elements; as most glass lenses introduce only positive dispersion, the results are additive and can become severe. Similarly, head-mounted multiphoton systems that require a long fiber-optic cable for beam delivery will generate a lot of dispersion (unless specialized fibers are used)^43,44^.

The broadening effect can be counteracted with a process called dispersion compensation, where the user purposefully introduces a fixed magnitude of dispersion that exactly matches that of the microscope’s optical path but of the opposite sign, such that the combined dispersion from the compensation unit and microscope sums to zero net dispersion. Some lasers have dispersion compensation included as an integral component option (for example, Coherent Vision and Axon, Spectra Physics Mai-Tai DeepSee and others). For lasers that do not include dispersion compensation, either a commercial or home-built external dispersion compensation unit is used or, in many cases, no compensation system is present, and the user has limited opportunity to modify the pulse width but should still consider the role dispersion may play in their system.

For two-photon microscopy, the main consequence of temporally stretched pulses is that the effective two-photon excitation efficiency drops. The drop in efficiency for nonlinear processes is often detrimental, even though the total average power being delivered to the sample remains the same. For higher-order processes, such as three-photon excitation, the falloff in signal with longer pulse widths is even more rapid. For shorter pulses (≤50 fs), it is especially important to note that the complete ‘dispersion’ relationship also includes higher-order terms that may need to be controlled as well^45,46^.

### Photomultiplier tube performance

Most multiphoton microscopes use one or more PMTs to detect light. In brief, the objective and collection optics direct photons emitted from the sample to the primary photosensitive element of the PMT, its photocathode. Photons having sufficient energy will cause a photoelectron to be generated via the photoelectric effect and then amplified through a cascade of dynodes (involving a high potential difference distributed across a chain of increasingly positive dynodes). This converts single photoelectrons to a much larger number of electrons at the PMT anode (~10,000–100,000). The bolus of charge arrives at the anode over ~5–10 ns, yielding a small burst of current. Typically, a transimpedance amplifier (TIA) is then used to convert this photocurrent to an analogue voltage and amplify that further. That voltage signal is then digitized by a high-speed analogue to digital converter (ADC) card in the acquisition system.

It is important to acknowledge that PMT performance, particularly for the more sensitive GaAsP detectors, degrades over time. Therefore, the PMT should ultimately be regarded as ‘consumable’ and be replaced periodically if the microscope’s detection performance is to be maintained. Most often, degradation is related to the total charge that has passed through the PMT, and this leads to a loss of both cathode sensitivity and photocurrent amplification but can also manifest as an increase in dark current and a decrease in dielectric resistance^47^. Moreover, ‘day 1’ performance, as well as rate of decline, vary substantially across units of the same PMT model and the rate of deterioration is influenced by usage (for example light exposure and average anode current).

A quantitative means to characterize PMT performance is, therefore, useful to (1) select between different units when installing a new PMT into the microscope, (2) diagnose issues that might arise with image quality and (3) benchmark performance over time to make informed decisions about when to replace a PMT.

We note that a laboratory’s specific performance requirements and financial considerations will also play into decisions around when to replace PMTs. However, by quantifying performance, a consistent policy can be adopted that, alongside other routine tests and maintenance described in this protocol, should allow minimum standards of data acquisition to be maintained.

### Estimating absolute magnitudes of fluorescence signals

The overall acquired fluorescence signals are influenced by factors such as laser power, focus, properties of the fluorescent indicator, light scatter and overall detection efficiency. It is imperative to understand and control each factor. Mismanagement of any of these factors can lead to a substantial decrease in signal intensity.

Fluorescence signals are commonly expressed in arbitrary units or on relative scales, such as the d*F*/*F* ratio (the amplitude of fluorescence change relative to the baseline fluorescence level), masking any degradation in signal magnitude. As a result, two laboratories following similar imaging protocols may record vastly different signal strengths for similar measurements, such as somatic calcium signals, without realizing the discrepancy. Low signal magnitudes lead to noisier, less precise measurements. However, without a method to evaluate quantitative signal magnitudes that could allow a laboratory to detect and address imaging system problems, laboratories may instead attempt to only compensate for decreased magnitudes of signals through post processing, missing opportunities for improving the primary data.

We suggest reporting fluorescence signals in absolute physical units such as detected photon counts per second—instead or in addition to simply reporting the relative d*F*/*F* signal. This standardized method offers a consistent and clear way to demonstrate signal levels, making it an invaluable tool for longitudinal system performance monitoring and simplifying comparisons between different imaging systems.

Direct photon counting, while feasible, is currently rare in multiphoton microscopy, as it requires specialized electronics^48^. However, it is possible to use signal–noise statistics to accurately estimate the detector photon sensitivity and translate detected signals into estimated photon counts. Photon count estimation is well established in photon-limited imaging modalities such as radiography^49^. Prior multiphoton studies have included variations of this procedure in their analysis^50–52^. However, the method has not yet been applied uniformly across laboratories to establish quantitative benchmarks. Here, we provide a procedure for estimating photon sensitivity, the photon flux (photon counts per unit area per unit time) and photon rates (for example, photon counts from an entire cell per unit time). We also provide a guide for interpreting these results. This approach offers a quick and intuitive way to evaluate imaging performance. However, it is important to recognize that it does not provide a detailed understanding of specific problem sources within the system. While the method works best on static fluorescent slides, it can work well even in the presence of physiological signals, although care must be taken to recognize and isolate the quantum noise. The consistent application of this absolute metric can lead to universally accepted standards for recorded signal quality, fostering precise expectations and enhancing reproducibility across the scientific community.

### Advantages and limitations

These protocols are intended as a practical resource for the multiphoton imaging community. This is not meant to be an encyclopedic resource on the theory, design or complete optical and detection characteristics of the instruments. We are purposefully focusing on measurements that can be performed by users with turn-key systems, that is, those without advanced optical metrology equipment. For example, we are not including a procedure for measuring the pulse shape and spectral properties, which would require an expensive optical device (an autocorrelator), as well as extensive optical expertise to use it properly. Therefore, this protocol is not a complete characterization of all aspects of multiphoton microscopy hardware relevant for designing such a system. In short, this is not a ‘build’ manual, as there are already many such excellent resources, such as refs. 14,15,53–55, as well as many books, articles and publications that include information more directly relevant to either designers or to readers interested in historical background or practice^2,14,16–18,21,29,56–61^.

We have executed these protocols on many different multiphoton systems and made the step-by-step instructions as generic as possible; however, it is still possible that some measurements may be difficult to perform on some systems, depending on software control limitations.

### Alternative methods

Alternative characterization efforts are underway; for example, the QUAREP-LiMi initiative (https://quarep.org/) that aims to improve quality assessment and quality control for light microscopy. This effort, with widespread community support from scientists and manufacturers, has already produced protocols for general light microscopy^62–64^. Our goals are different in that our narrower focus is meant to provide a single, comprehensive characterization of a multiphoton microscope.

There are many protocols on various aspects of quality control for confocal microscopes, many of which use similar scanning optics to those used in multiphoton microscopes (PSF only^63,65^, quantification^66^ and reporting^67^). Our effort is also similar to a recent protocol on ‘Strategic and practical guidelines for successful structured illumination microscopy‘^68^. These protocols do not cover key issues specific to the multiphoton case, however, see recent publications regarding laser assessment^45,69^, as well as earlier multiphoton-centered literature^56,70^. For the nonlinear excitation that enables multiphoton microscopy, pulsed laser sources are required and, thus, additional factors, such as laser pulse repetition rate, pulse width and compensation for pulse broadening, require consideration. Additionally, test samples must undergo efficient two-photon excitation without bleaching or burning, which makes some quantifications particularly challenging. Finally, it is important to characterize multiple aspects in a coherent fashion, so our goal is to have a collection of related procedures in one protocol. The methods presented were chosen by the authors based on cost, ease and practicality constraints. A detailed review of competing approaches is beyond the scope of this protocol.

## Materials

### Software

- Software that can manipulate images (ImageJ/FIJI, MATLAB, Python/Napari)
- Software that can plot data (MATLAB, Python)
- Microscope control software that allows live image histogram viewing

### Equipment and reagents

- Laser scanning multiphoton microscope system (e.g. Ultima 2Pplus, Bruker; Bergamo III Series, Thorlabs)
- **▲ CRITICAL** Keep a shared, dated log of all changes to the microscope system (for example, alignment, calibrations, measurements, software updates and so on) that is easily accessible by all users. A good ‘rig log’ pays dividends in years to come.
- **▲ CRITICAL** For spatial resolution measurements there must be a motorized vertical translation stage with ≤0.5 µm minimum incremental movement
- Pulsed laser suitable for multiphoton excitation (e.g. Mai Tai HP DS, Spectra Physics; FemtoFiber ultra 920, Toptica; Chameleon Vision II, Coherent) with power modulation device built in or separately installed after the laser (e.g. Pockels Cell Model 350-80LA Electro-Optic Modulator and Model 302RM Driver, Conoptics)
- Objective lens(es) suitable for multiphoton microscopy (e.g. CFI75 LWD 16X W, Nikon)
- Lens cleaning tissues (e.g. MC-5, Thorlabs)
- Deionized water
- Mechanical pipettes and tips (e.g. F167360, Gilson for 1–1,000 µL volumes)

### Procedure 1: laser power at the sample

- Laser power meter (e.g. PM100D, Thorlabs)
- Power sensor head for laser power meter (e.g. S405C or S121C, Thorlabs; varies depending on application, see below)
- **▲ CRITICAL** Time-averaged laser power is measured with either a photodiode or thermal power sensor. Photodiodes convert light directly into an electrical signal, whereas thermal power sensors convert the thermal energy deposited by the light into a measurable voltage. Thermal power sensors are often recommended because photodiode sensors can saturate depending on the peak powers of your laser, though thermal sensors are slower to respond to power changes and more sensitive to external conditions. The wavelength range, power range, sensor area and sensor resolution should all be considered when choosing a power sensor. The wavelength and power range should match the laboratory’s imaging needs; the sensor area should be larger than the exit area of your objective to allow sufficient light to get to the active sensor area, and the sensor resolution must be fine enough to give you the accuracy you need.
- Clamping hardware (bolts and clamps, e.g. CL3/M and SH6MS12, Thorlabs) or adhesive (tape or sticky tack; e.g. 134-7321 or 342-4500, RS components) for securing the power sensor to the microscope stage

### Procedure 2: FOV size

- Ethanol (e.g. 1009711000, Sigma-Aldrich)
- Gridded microscope slide (e.g. R1L3S1P, Thorlabs; 57-877, Edmund Optics)
- Autofluorescent plastic slide (e.g. 92001, Chroma)
- Mechanical clamp (e.g. 549-274, RS Components), adhesive (tape or sticky tack; e.g. 134-7321 or 342-4500, RS components, sticky tack) or epoxy resin (132-605, RS Components)
- Glass microscope slide (e.g. MS10UW2, Thorlabs)
- Nail varnish
- Coverglass (e.g. CG15KH1, Thorlabs)
- Copper grid used for electron microscopy (EM; e.g. 2145C-XA, SPI supplies). These grids have a pitch of 25 µm with 19-µm-wide square holes. Dry copper EM grids will emit light when stimulated by a multiphoton laser

### Procedure 3: FOV homogeneity

- Autofluorescent plastic slide (e.g. 92001, Chroma)
- Fluorescein (e.g. 46960-25G-F, Sigma-Aldrich)
- Petri dish (e.g. PET2022, Scientific Laboratory Supplies Ltd)
- Delicate single-ply wipes (e.g. 7551, Kimberly-Clark Professional)
- Epoxy resin (132-605, RS Components)

### Procedure 4: spatial resolution

- Bench-top microcentrifuge (e.g. CLS6770, Sigma-Aldrich)
- Heated plate (e.g. Z742547, Sigma-Aldrich)
- Coverglass of known thickness (e.g. CG15KH1, Thorlabs)
  **▲ CRITICAL** Keep the thickness constant between repeated measurements. Further discussion can be found in Box 4.
- Microcentrifuge tubes, 1.5 mL (HS4323-500EA, Sigma-Aldrich)
- Low melting point agarose (16520050, Invitrogen)
  **▲ CRITICAL** Low melting point agarose is recommended for the ease of sample preparation.
- Beads, 0.2 µm (F8811, Invitrogen)
  **▲ CRITICAL** Choose beads with excitation peaks that match the laser wavelengths routinely used for experiments
- Glass/microscope slide with a concavity or well in the center (632200, Carolina, or MS15C1, Thorlabs)
- Vortex mixer (e.g. CLS6776-1EA, Sigma-Aldrich)

### Procedure 5: group delay dispersion optimization

- Pollen grain slide, mixed pollen grain slide (304264, Carolina Biological Supply Company)
- Fluorescein (e.g. 46960-25G-F, Sigma-Aldrich)
- Autofluorescent plastic slide (92001, Chroma)

### Procedure 6: photomultiplier tube performance

- Tritium/phosphor vial 3 mm × 11 mm (T56041I, EDCGEAR or T311, mixglo.com)
- SM1 lens tube, 0.5 inch (SM1L05, Thorlabs)
- SM1 end cap (SM1CP2M, Thorlabs)
- Epoxy resin (132-605, RS Components)
- Mounted Pinhole, 500 µm (P500K, Thorlabs)
- (Optional) Neutral density filter (NE10B-A, Thorlabs)
- (Optional) Narrow bandpass filter (varies depending on configuration, e.g. ET520/20m, Chroma Technology)

### Procedure 1: measuring laser power at the sample

**• TIMING 0.5-2 h**

**▲ CAUTION** Ensure that local laser safety rules are always followed to prevent fire and eye/skin damage. Consult a laser safety officer for advice.

**▲ CRITICAL** Some steps have multiple possible methods for achieving the result, ensure to maintain consistency when measuring the beam in future iterations of the procedure by recording your decision at each step (Box 1).

**Fig. 3:**
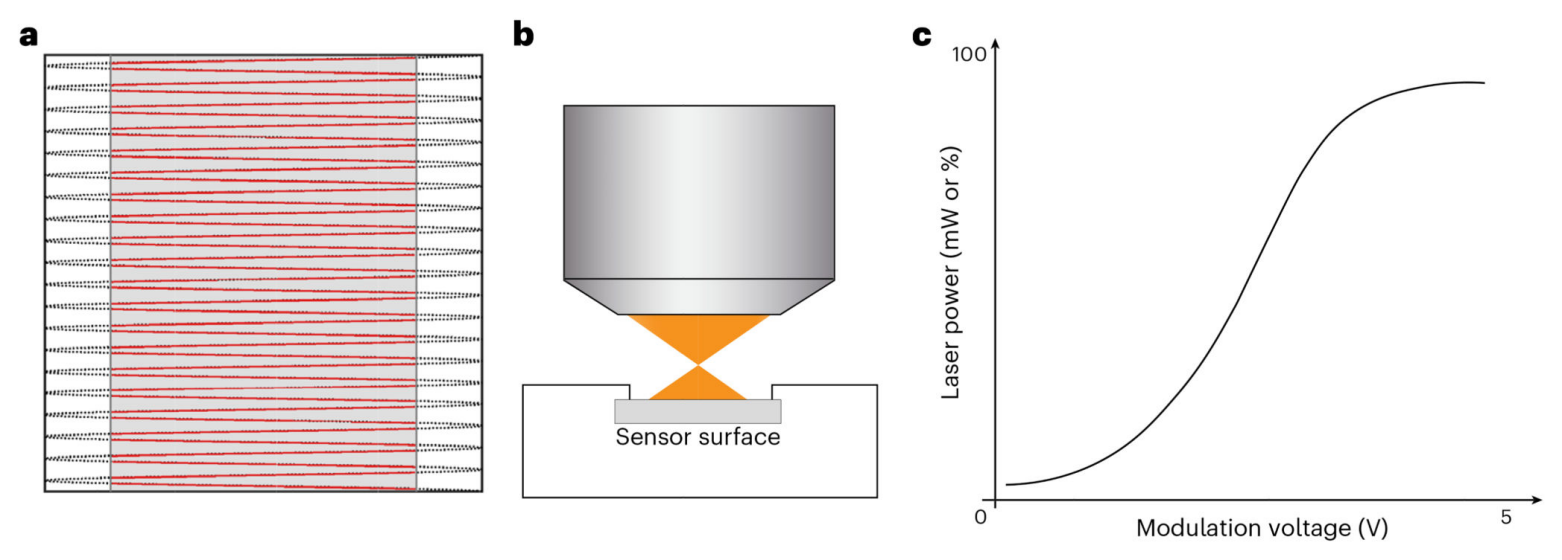
Laser power measurement. **a**, The path of a resonant-scanned focused laser beam over a sample. The beam moves sinusoidally along the fast axis (*X* in this case) whilst being scanned in orthogonal *Y* direction with a linear galvo. The area over which the beam moves is known as the scan field. On the left and right edges, the beam slows as the scanner changes direction and turns around. In these areas the potential for photodamage is greatest, as the beam is traveling more slowly over the sample. Thus, the beam is typically ‘blanked’ or disabled during these epochs. In a resonant scanning microscope, the beam is usually blanked ~30% of the time. The image field (red lines and gray region) is the area over which the beam is on and capable of exciting fluorescence. The dotted lines indicate the blanked turn-around regions. A power meter cannot distinguish these states and so returns a time-averaged power value over the whole scan field if the microscope is scanning during a measurement. **b**, Position of the power meter head with respect to the laser beam exiting the objective. The sensor surface should be close but must not be at the working distance of the objective lens, as the focused beam may damage the sensor surface leading to unreliable measurements and permanent damage to the sensor. **c**, An example laser power calibration curve. Output power can be represented as a percentage of total available laser power or in direct power units (mW), depending on microscope configuration. The purpose is to create a lookup table that allows linear adjustment of power on the edges of the modulation range for modulation devices (such as Pockels cells) that have nonlinear response.

1. Turn on the microscope hardware and software necessary for controlling laser beam scanning and laser shutters.
2. Turn on the laser, and if tuneable, select the wavelength normally used for imaging applications.
3. (Optional) Some laser power modulators, such as Pockels cells, are temperature sensitive. If using such a modulator, open the required shutters so that the beam passes through the device to warm it to an equilibrium temperature. In an ideal setup, there will be an additional hard shutter downstream of the laser power modulator, and this shutter is used to prevent light from entering the microscope when the beam is ‘off’.
4. Wait for the laser power output to stabilize. The time taken for stabilization depends on the laser and can be measured and used in future iterations of this procedure. For the first time, wait at least an hour.
**◆ TROUBLESHOOTING**
5. (Optional) If using a Pockels cell, ensure the bias voltage is set to the value when it was last aligned, and keep a log of that number somewhere.
6. Install an objective lens into the microscope that you want to measure the laser power for.
  ▲ **CRITICAL STEP** Ensure the objective is clean by looking through the bottom of the objective in the room light to magnify the front lens. If it is not, clean the objective lens using a suitable method depending on the type of objective and the contamination. Consult manufacturers recommendations if unsure of the procedure.
7. Plug the power meter sensor connector firmly into the console.
8. Turn on the power meter.
  **▲ CRITICAL STEP** Ensure the battery is charged or it is plugged into a power source to last for the duration of the measurements.
9. Set the power meter console measurement wavelength to match the laser wavelength being measured.
10. Place the sensor under the objective and secure the sensor in place using a suitable method for your stage configuration (bolt/clamp or adhesive).
  **▲ CRITICAL STEP** Center the objective over the active area of the sensor laterally and set the height of the objective such that the laser will not be focused on the power meter’s sensitive surface. It is a balancing act to set the objective-sensor distance. The sensor should be close to but not at the objective’s working distance, as this will ensure the sensor captures all light while also minimizing the chance of damaging it with a tightly focused beam (Fig. 3b). More consistent readings will be obtained if the beam fills a large proportion of the sensor, but if light falls outside of the active area, an inaccurate result will be obtained.
11. (Optional) Apply appropriate immersion media for the objective if the power sensor is designed for it.
12. ‘Point’ or ‘center’ the beam in the software so it is stationary in the center of the FOV. If this is not possible, a similar result is achieved by zooming in by a factor of 10× or 20× and scanning a small area with beam blanking disabled (Fig. 3a).
13. ‘Zero’ the power meter to set the reading to 0 mW and remove any background offset.
14. Open any remaining shutters so that the beam passes through the system to the sensor.
15. Find the minimum power by adjusting the software’s power modulation control until a minimum reading is seen on the power meter.
16. Record the power meter measurement (usually in units of mW) and the power modulation value in the software (that is, percentage, volts or arbitrary units) for this minimum power.
**◆ TROUBLESHOOTING**
17. Find the maximum power by adjusting power modulation until a maximum reading is reached.
18. ▲ **CAUTION** Do not exceed the power sensor’s upper power limit, otherwise the powersensor surface could become damaged and result in inaccurate readings.
19. Record the power meter measurement and modulation value for this maximum power.
20. Record intermediate laser powers and modulation values that are commonly used in your experiments.
  **▲ CRITICAL STEP** It is common for modulators to respond nonlinearly to the control input so laser power can be a sigmoidal function of the percentage power value, where the maximum power may occur well before ‘100%’ laser power (Fig. 3c).
21. Arrange the above power meter measurements and software modulation values in a table of a suitable spreadsheet program for future comparisons.
  **▲ CRITICAL STEP** Provide information regarding how the reading was made (stationary or scanning beam), and if scanning, report the temporal fill fraction. If you have access to different power meters, report the model number or serial number of the sensor head.
22. Repeat Steps 9–20 for any other wavelengths that are important for imaging experiments.
23. Compare new measurements to historical power measurements and ensure that the laser power is not changing.
**◆ TROUBLESHOOTING**

### Procedure 2: measuring FOV size

**• TIMING 2 h**

**Fig. 4:**
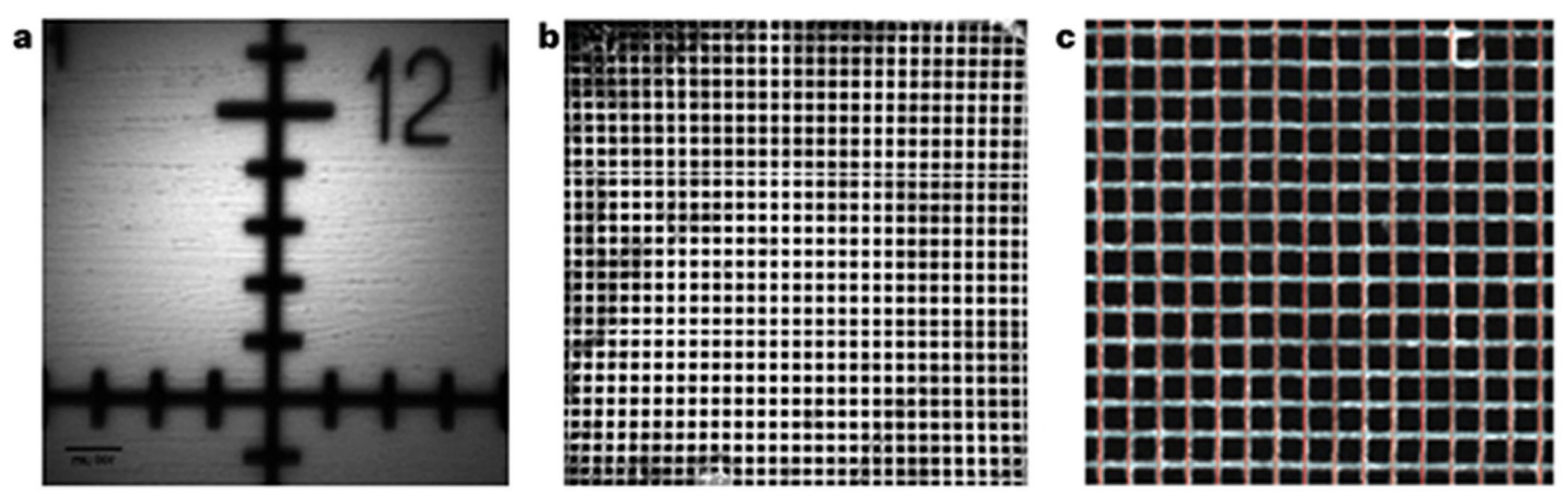
FOV size measurement. **a**, Two-photon image of a 1 mm grid with 100 µm divisions for the maximum scan angle of the microscope system (that is, the minimum zoom). Here, the FOV size is ~800 µm. **b**, An image of a 25 µm copper EM grid. This image shows the ~1,200 µm FOV and displays minor pincushion distortion at the left and right edges (note how the vertical line on the right and left sides bows in from the vertical line defined by the edge of the image). **c**, Grid lines are detected and overlaid on top of the EM grid image using the ‘MicsPerPixel’ software tool^71^.

**Fig. 5:**
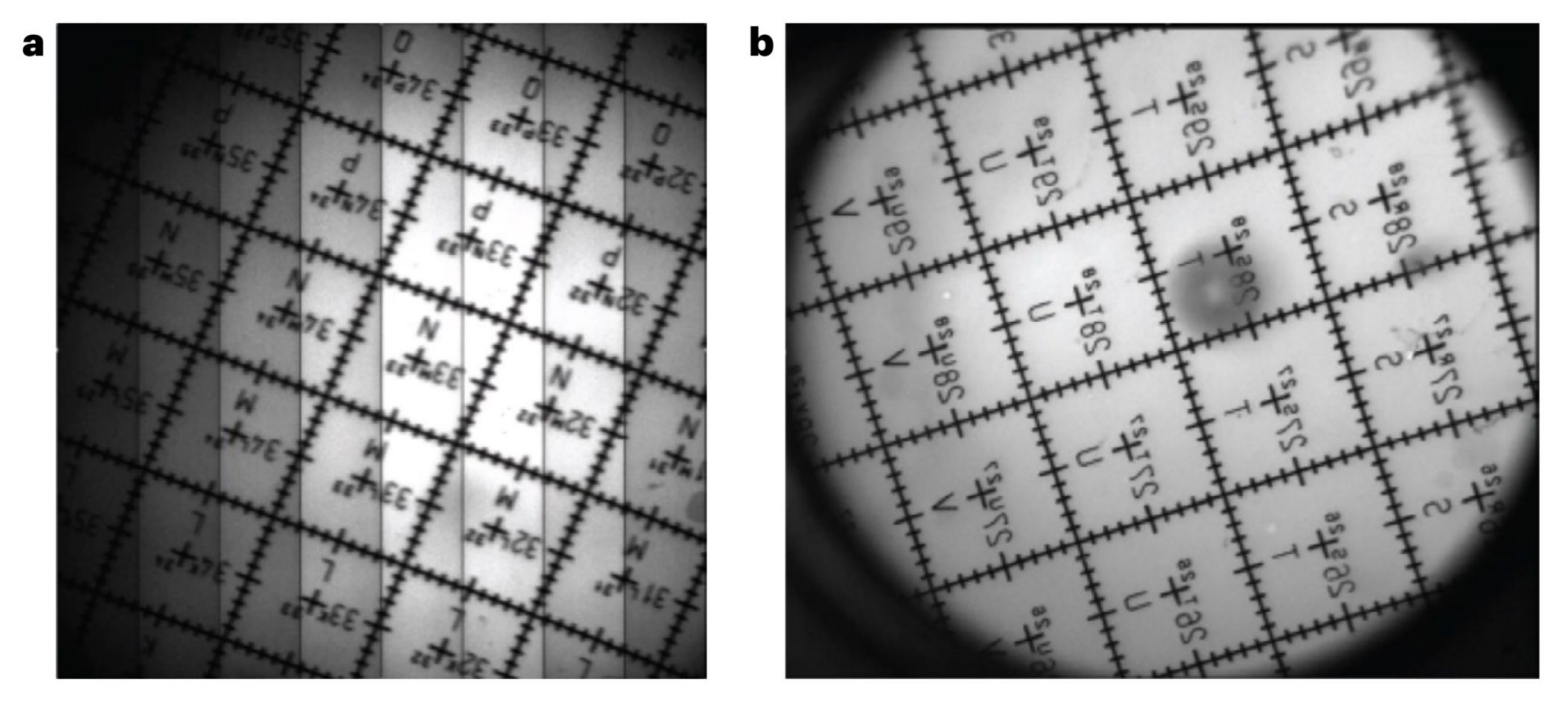
FOV size comparison for two-photon and epifluorescent modes on a large FOV microscope (Mesoscope). **a**, Tiled two-photon image of the Mesoscope FOV (~5,000 µm). **b**, Epifluorescent image of the Mesoscope FOV showing a ~45° rotation and vertical reflection, compared with the two-photon image.

1. Turn on the microscope hardware and software necessary for controlling laser beam scanning.
2. Turn on the laser and wait for the power to stabilize (see Procedure 1). Select the wavelength normally used for imaging applications.
3. Prepare the relevant calibration sample (see Box 2) for FOV size measurements according to the equipment you have:
  (A) **(Optional) Prepare a gridded slide**
    (i) Clean a gridded microscope slide and an autofluorescent plastic slide using single-ply wipes and ethanol.
    (ii) Place the autofluorescent plastic slide flat on a bench.
    (iii) Identify which side of the gridded slide has etchings.
    (iv) Add a droplet of water onto the autofluorescent plastic slide, and place the gridded slide such that the etchings face the autofluorescent slide.
    (v) Clamp the two slides together with a mechanical clamp, adhesive or epoxy resin.
  (B) **(Optional) Prepare a copper grid**
    (i) While keeping the grid dry, place it on a conventional glass slide or an autofluorescent slide (for a bright negative image of the grid).
    (ii) Place a coverslip over the top of the grid.
    (iii) Seal the coverslip with nail varnish.
4. Install an objective lens that you routinely use for imaging in to the microscope.
5. Place the sample under the objective lens.
  **▲ CRITICAL STEP** Place the sample perpendicular to the optical axis to eliminate any tilt with respect to the imaging plane.
  (Optional) It is possible to use the microscope’s laser beam to align the sample to the objective and reduce tilt. For this, lower the laser power such that no more than a few milliwatts exits the objective. Close any iris in the excitation path to only let through the very center of the beam. Make sure the beam hits the sample and then use an infrared (IR) viewing card and an IR viewer to visualize the reflected beam. Here, the reflection will be very weak, on the order of 1–5%, so an IR viewer is needed to visualize it. Adjust the sample tilt such that the reflected beam propagates directly along the incident beam.
6. Apply any immersion media appropriate for the objective lens and position the lens at roughly its focal distance away from the sample.
7. Focus on the calibration sample gridded surface.
  (Optional) If using a fluorescent slide overlaid with a grid, image the very surface of the fluorescent slide to get a negative image just underneath the grid (Fig. 4b).
8. Acquire an image of the calibration sample at the minimum zoom to give the largest FOV (Figs. 4a and 5a) (Box 3).
  (Optional) If the system has field curvature or a large FOV (>1 mm), it might be difficult to obtain a completely flat image of the sample. In this case, it is necessary to acquire a *z*-stack that covers the axial extent of the tilted imaging plane and make a maximum projection of that stack for the next steps.
9. Record the number of whole grid squares required to cover the width and height of the FOV. ▲ **CRITICAL STEP** If the sample is rotated so the grids do not run horizontally or vertically, rotate the sample to match the grid rows/columns with the *X* and *Y* axis of the image.
  (Optional) As counting the lines ofa copper EM grid is time consuming, using an automated tool may be preferable (Grid2MicsPerPixel tool^71^) (Fig. 4c).
10. Record the total number of pixels required to cover the same distance as in Step 9.
11. Multiply the number of grids from Step 9 by the grid pitch size in microns to give the total width and height in microns.
12. Divide the distance in pixels from Step 10 by the distance in microns from Step 11 to get the micron-to-pixel conversion and multiply that by the total pixels in the image to get the FOV size.
13. Evaluate whether there is pincushion or barrel distortion^72^ in the images (Fig. 4b). The presence of these aberrations would signify an off-axis optical performance degradation in the system.
**◆ TROUBLESHOOTING**
14. Repeat Steps 4–12 for any other objectives used for imaging, as calibration is only relevant for the objective used to do that calibration.
  **▲ CRITICAL STEP** Calibration files are usually saved for each objective in the acquisition software so that the pixel size is calibrated for every acquired image without any manual metadata editing.
15. (Optional) If the microscope has other imaging paths, repeat the measurement for one objective using these imaging modalities (for example, epifluorescence or brightfield) (Fig. 5a,b) and note any rotation/mirror effects between the two imaging modes, which will be the same for all objectives.

### Procedure 3: assessing FOV homogeneity

**• TIMING 2 h**

**▲ CRITICAL** This procedure describes how to assess the homogeneity of the FOV by imaging a uniform fluorescent sample and measuring how the image intensity varies across the image. The resulting measurements are a description of the overall system’s performance, meaning that any dropoffs in intensity could be caused by optical degradation in either (or both) the excitation and collection paths.

**Fig. 6:**
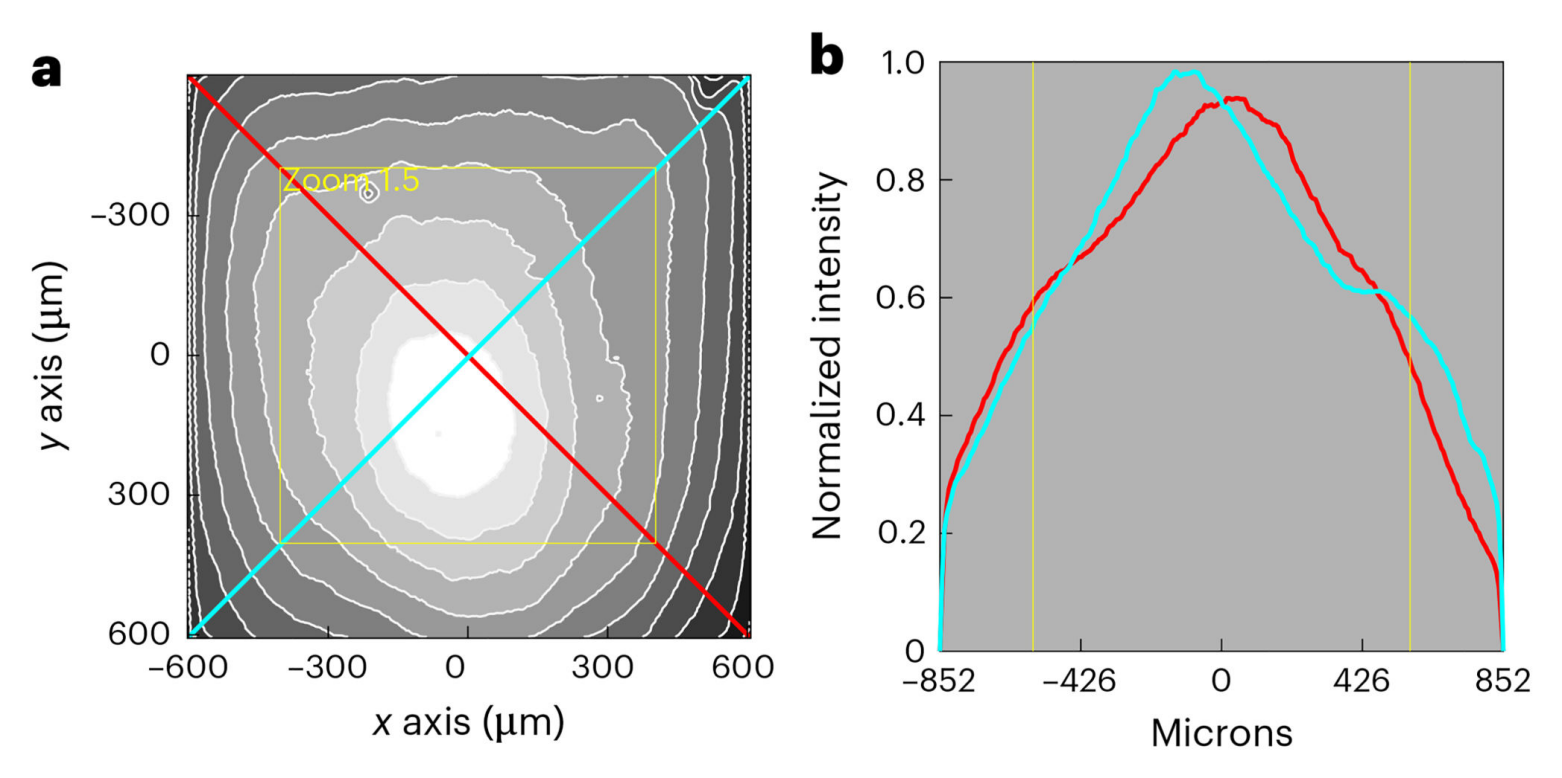
FOV homogeneity. **a**, Homogeneity calibration image of the uniform fluorescent slide acquired using a 16× objective at maximum scan angle on a system that allows for large scan angles. Homogeneity dropoff profiles and different zoom factor overlays are shown (different systems may have different scan areas for the corresponding ‘zoom’ factor). The area of peak brightness is offset downward slightly in the *y* axis. This indicates a possible misalignment in the optics. The dark spots arise from imperfections in the slide surface. **b**, Intensity profiles along the diagonals (cyan and red) demonstrate nonuniformity of excitation at the maximum zoom factor.

1. Turn on the microscope hardware and software necessary for controlling the laser beam scanning.
2. Turn on the laser and wait for the power to stabilize (see Procedure 1). Select the wavelength normally used for imaging applications.
3. Install an objective lens that you routinely use for imaging in to the microscope.
4. Prepare a thick uniform fluorescent sample, either an autofluorescent plastic slide or a fluorescein bath (if using a water-dipping objective) with 1 µL of fluorescein to 10 mL of water inside a suitable petri dish.
  **▲ CRITICAL STEP** Many objectives used for in vivo multiphoton imaging have field curvature, where the imaging plane is not actually a plane but is instead shaped as a shallow bowl (see Procedure 2 to measure this). This is particularly noticeable for some large FOV systems^42,73,74^. To eliminate the effect of small field curvature on field homogeneity, the sample for this measurement should be sufficiently thick that the beam remains in the sample across the entire field.
5. Place the sample under the objective lens and focus on it (dip water immersion lenses directly in the fluorescein).
6. Set the laser power to <5 mW (see Procedure 1 to measure this).
7. Begin imaging and turn the PMT gain up from 0 until the signal from the sample is seen.
  **▲ CRITICAL STEP** The PMT gain should be turned up slowly to avoid damaging the PMTs with a potentially very bright sample. If no signal is seen, adjust the objective focus up and down to search for signal from the sample before turning up the PMT gain further.
8. (Optional) If using a fluorescent slide, find the sample surface then slowly lower the objective until all hints of the surface inhomogeneities have vanished. You will probably need to focus ~100–200 µm deep.
9. Acquire an image of the sample using the largest FOV size the system allows for.
  **▲ CRITICAL STEP** The width/height of the image in pixels needs to be sufficient to sample the inhomogeneity. Additionally, an average of multiple images is advisable to reduce noise.
10. (Optional) If this is the first time you are performing the measurements, acquire more images using higher zooms (smaller FOVs) at 50% and 25% of the full FOV, for example. This will help diagnose uncommon inhomogeneities that could arise from the laser power modulator.
**◆ TROUBLESHOOTING**
11. (Optional) If the saved image is not already scaled correctly, translate the pixels to microns using the FOV size calculated in Procedure 2.
12. Normalize the image by dividing all pixel values in the image by the maximum pixel value.
13. Plot a line profile through the horizontal/vertical axes and through the diagonals of the FOV (Fig. 6a). The horizontal/vertical profile is informative about each scan axis (from the *X* or *Y* scanner) (Fig. 6b).
14. Evaluate how symmetrical the brightness across the FOV is. Because of the radial symmetry of most optical systems, a well aligned microscope should have the brightest part of the field in the center of the image and the brightness should fall off evenly around the center.
**◆ TROUBLESHOOTING**
15. If previous measurements have been made, refer to them to determine if there have been any changes in homogeneity that need addressing.

**◆ TROUBLESHOOTING**

### Procedure 4: measuring spatial resolution

**• TIMING 3.5 h**

**Fig. 7:**
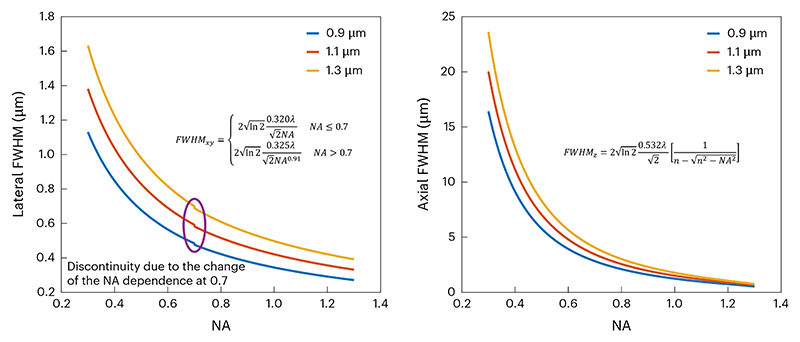
Two-photon FWHM as a function of NA and wavelengths. The formula is adapted from Zipfel et al.^21^. NA is the numerical aperture of the objective lens. In FWHMz, *n* is the RI of the medium where the sample is embedded and set as water in this plot. The RI of the water, *n*, is wavelength dependent and is [1.328, 1.3255, 1.3225] at [0.9, 1.1, 1.3] µm, respectively.

**▲ CRITICAL** As the resolution is a function of wavelength, the measurements should be performed with excitation/emission wavelengths close to those used in experiments (Fig. 7). Lens designs, antireflective coatings, dichroic filters and mirrors all have wavelength-dependent properties and can influence the measurements made.

**Fig. 8:**
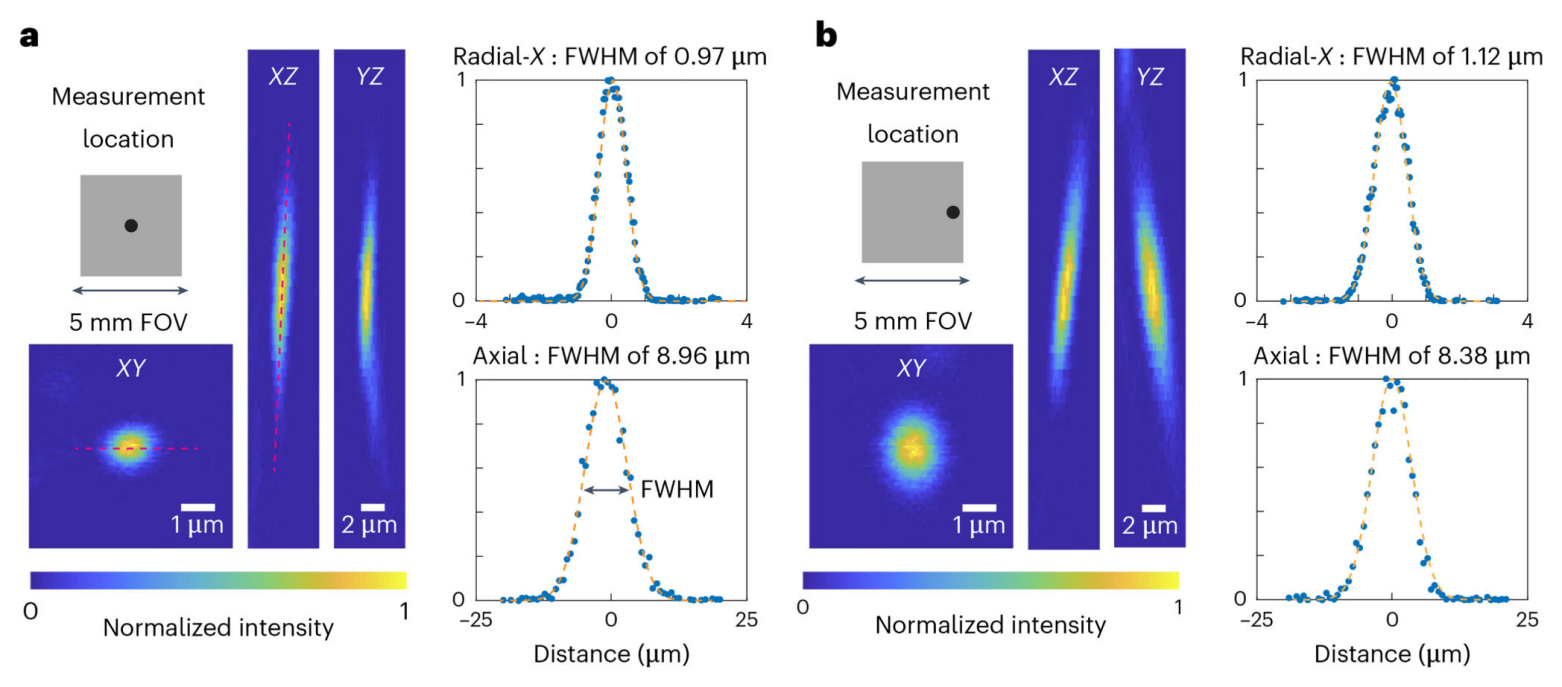
Example measurement of PSFs. **a**,**b**, Fluorescent beads (0.2 µm) were embedded in 0.75% (wt/vol) agarose gel; 40 µm *z*-stacks were acquired at the depth of 500 µm, and beads at the center (**a**) and the edge (**b**) of the FOV were measured. The example images are shown from the *XY, XZ* and *YZ* cross sections, respectively. The intensity profiles of the beads (red dashed lines) in the *X* direction on the *XY* plane and in the *Z* direction are plotted, which are fitted to a Gaussian curve (orange dashed line) to extract the radial-*X* and axial FWHM of the PSF (note the PSF degradation for the lateral position of the bead).

1. Turn on the microscope hardware and software necessary for controlling laser beam scanning.
2. Turn on the laser and wait for the power to stabilize (see Procedure 1). Select the wavelength normally used for imaging applications.
3. Prepare the bead slide (Box 4):
  (a) Make a 0.75% (wt/vol) agarose solution by dissolving 0.150 g low melting point agarose in 20 mL water at ~75 °C.
  (b) Vortex the agarose solution until completely dissolved.
  (c) Add 1,000 µL of the agarose solution to a 1.5 mL microcentrifuge tube and wait for the agarose to cool slightly to 50 °C.
  (d) Add 0.2 µL of 0.2 µm beads to the 0.75% (wt/vol) agarose solution to reach a dilution of ~1:5,000 in volume.
  (e) Vortex the mixture for 5 s and then spin it down for 5 s with a bench-top centrifuge to make the mixture uniform and remove bubbles.
  (f) Transfer the bead/agarose mixture into the concavity of a welled glass slide and cover with a coverglass of known thickness.
    **▲ CRITICAL STEP** Avoid bubble formation during the process and completely fill the space between the slide and the cover glass with the mixture, leaving no air gap. Air gaps and bubbles can move around during the imaging session and cause movement of the beads.
  (g) Wait for 15 min for the mixture to completely cool down and solidify.
    **▲ CRITICAL STEP** (Optional) If planning to keep the slides for longer than 2 h, seal the sides of the coverglass (for example, nail polish, wax, optical glue and so on) to prevent evaporation.
4. Install an objective lens that you routinely use for imaging in the microscope.
5. Place the prepared slide under the objective lens on a motorized stage and use the imaging system to find the focal plane of the beads with a FOV size of 100–200 µm. ▲
  **CRITICAL STEP** The laser power should be close to the minimum required to clearly identify individual fluorescent beads. If higher powers are used, there is a risk of over- or underestimating the excitation volume, through either saturation or bleaching respectively, and this issue is exacerbated when using fluorescence molecules with relatively large cross sections^21^.
**◆ TROUBLESHOOTING**
6. Zoom in to a pixel size of ~0.04 µm and plot the intensity of multiple putative single beads and small clusters of beads as a histogram. This process will reveal a series of peaks where each peak will be a multiple of the intensity of a single bead. If sufficiently sampled, the interval between peaks will be regular and equal to the intensity of a single bead. Use this information to verify if a region of interest is a single bead.
7. Identify a single, isolated bead with a nonsaturating intensity.
  **▲ CRITICAL STEP** Ensure there are not any other beads in the axial scan range of the chosen bead, as this would interfere with the measurement.
  **▲ CRITICAL STEP** Additionally, the FOV should be large enough to ensure that the bead intensity returns to background levels, but no other beads should be present in that space.
  **▲ CRITICAL STEP** Examine the bead intensity and ensure the pixel intensity at any image plane does not saturate the PMT (that is, very few pixels reach the maximum possible intensity value of, for example, 65,536 for a 16-bit image). If that occurs, lower the laser power or the gain of the PMT or find another suitable bead.
**◆ TROUBLESHOOTING**
8. Acquire a *z*-stack of the bead with a 0.3 µm step size and appropriate total axial range.
  ▲ **CRITICAL STEP** Select the total range of the stack to be four to six times larger than the theoretical axial extent of the PSF. For example, for a system with an axial FWHM of 5 µm in theory, use 20–30 µm *z*-extent.
  **▲ CRITICAL STEP** The lateral pixel size and axial stage movement should be calibrated correctly using a structured sample (see Procedure 2 for lateral pixel size), rather than using the stage motor values.
9. Fit the intensity measurement with Gaussian curves in the lateral (*XY*) and axial (*Z*) direction, respectively (Fig. 8). We provide a tool^71^ for conducting such analyses in MATLAB. Another option is Python-based^75^ and can easily extract PSFs from multiple beads in a single image.
  **▲ CRITICAL STEP** In cases where the excitation volume is very tilted in *Z*, the Gaussian fit procedure should account for this tilt, otherwise, the excitation volume will be underestimated. The current version of our example code does not include a subroutine to correct the tilt. One could use a built-in rotation function, such as ImageJ (‘Image > Transform > Rotate…’) and MATLAB (‘imrotate’), to correct the tilt.
**◆ TROUBLESHOOTING**
10. Find the full width at half maximum (FWHM) of the curves in units of length (for example, nanometers or micrometers) and use these values to keep track of the resolution.
  **▲ CRITICAL STEP** When referring to a theoretical or expected resolution, be sure to include the equation, the values for any parameters in the equation and a paper reference, for example, Zipfel et al.^21^. Be clear whether FWHM, 1/e radius, or 1/e^2^ radius of the PSF is reported. This provides clarity to the reader and enables fair comparisons.
11. Repeat Steps 7–10 for three or more beads to achieve confidence in the resolution measurement.
12. Repeat Steps 7–11 for multiple locations in the FOV: the center, the edges of the *X* scan, the edges of the *Y* scan and different depths. It is useful to show where resolution breaks down. Instead of simply reporting the best values, show where the resolution starts to degrade and by how much. For routine checks, measuring at just two or three reference locations can be sufficient.
  **▲ CRITICAL STEP** It is recommended that beads are measured at different locations across the FOV (center and toward the edges) and at different depths. Optical aberrations tend to increase away from the center of the FOV, while SA and scattering vary over the imaging depth.
  **▲ CRITICAL STEP** Zoom in on beads at the edge of the FOV by offsetting the scan centre of the linear galvanometer scanner. This offsetting function might appear as ‘shift’, ‘offset’ or ‘park’ on the microscope control software.
  **▲ CRITICAL STEP** If an air immersion objective is used, due to the difference between the RI of the objective immersion medium (for example, air) and the specimen medium (for example, water), the actual focal position (Δfocus) within the specimen is moved a different amount from the stage movement (Δstage). Therefore, a correction factor is required to convert the axial stage movement to the actual focal movement. According to the reference^34^, a simplified calculation of the correction factor is
  (2) $$\frac{\Delta \;\text{focus}\;}{\Delta \;\text{stage}\;}=\frac{\text{tan}\left({\text{sin}}^{-1}\frac{\text{NA}}{2n_{1}}\right)}{\text{tan}\left({\text{sin}}^{-1}\frac{\text{NA}}{2n_{2}}\right)}=\frac{n_{2}\text{cos}\left({\text{sin}}^{-1}\frac{\text{NA}}{2n_{2}}\right)}{n_{1}\text{cos}\left({\text{sin}}^{-1}\frac{\text{NA}}{2n_{1}}\right)}=\sqrt{\frac{4n_{2}^{2}-{\text{NA}}^{2}}{4n_{1}^{2}-{\text{NA}}^{2}}},$$
  where *n*_*1*_ is the RI of the immersion medium, *n*_2_ is the RI of the sample and NA stands for the NA of the objective.

### Procedure 5: optimizing group delay dispersion

**• TIMING 1.5 h**

**Fig. 9:**
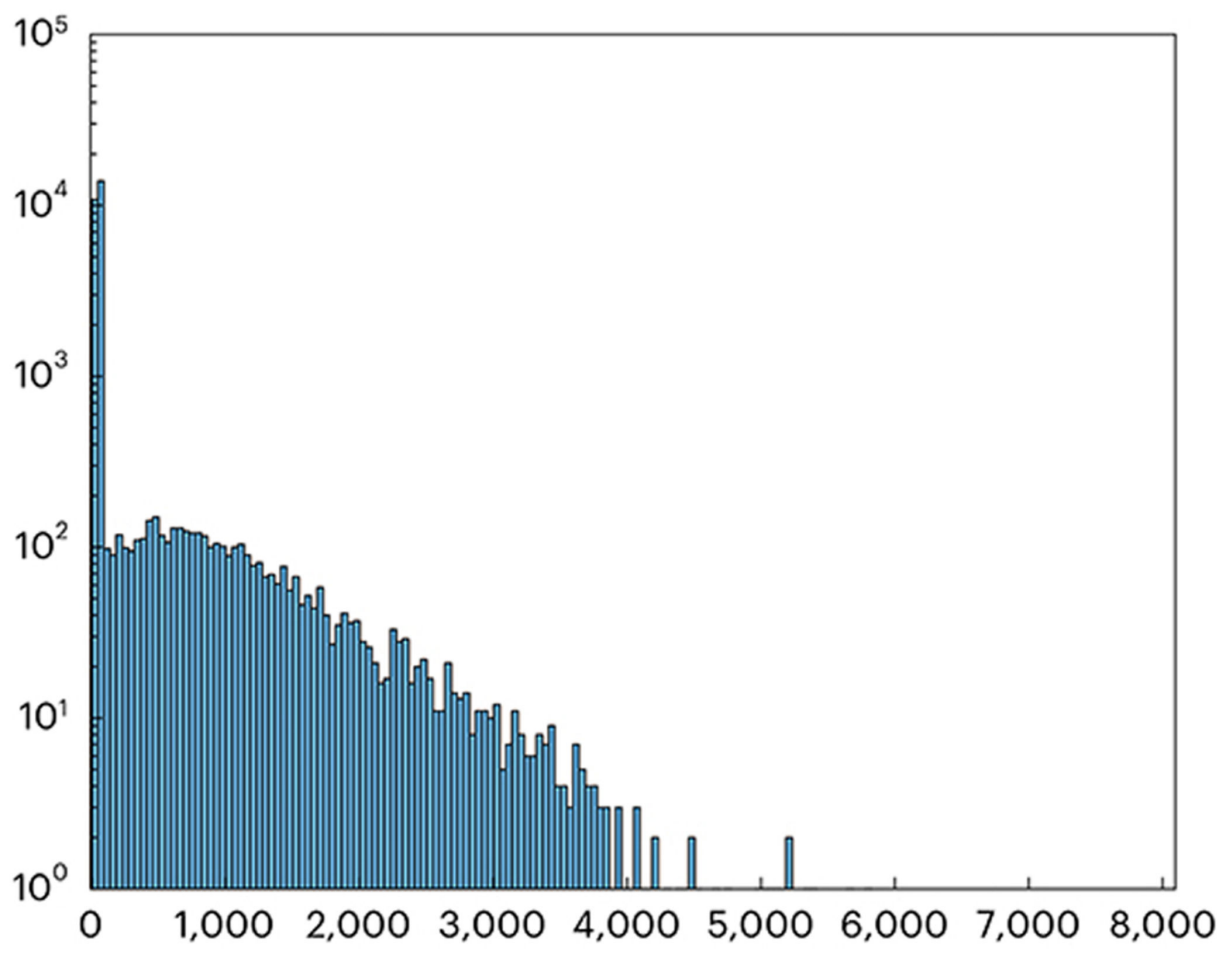
Example image pixel histogram. The pixel intensity value distribution is shown for one image frame while live scanning. The spread of this distribution is used to optimize laser pulse width.

**Fig. 10:**
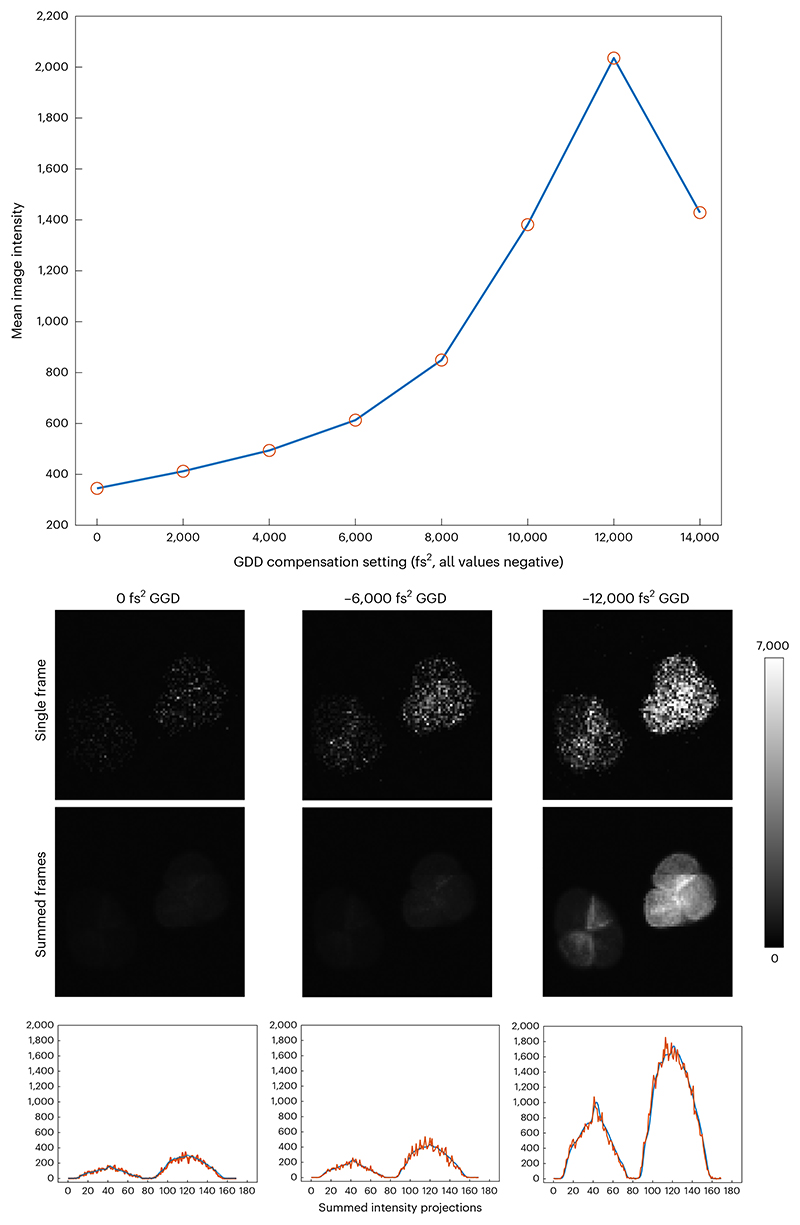
Pulse width optimization measurement. The top panel shows a plot of mean image intensity versus GDD compensation value for a fixed average laser power. For this system, ~-12,000 fs^2^ of compensation is necessary for the microscope to achieve highest excitation efficiency. The bottom panels show example images acquired of the different settings of GDD compensator and projected intensity plots that can be used for analysis described in the procedure.

1. Turn on the laser and wait for the power to stabilize (see Procedure 1). Select the wavelength normally used for imaging applications.
2. Turn on the microscope hardware and software necessary for controlling laser beam scanning.
3. Install a microscope objective lens used for imaging experiments.
4. Prepare a sample and place it under the objective lens, either:
  (a) For water immersion dipping objectives only, fill a Petri dish with a fluorescein bath of 1 µL fluorescein in 10 mL water.
  (b) A fluorescein drop under a coverslip, dilute 1 µL fluorescein in 10 mL water and pipette a 50 µL drop under the coverslip.
  (c) Autofluorescent plastic slide.
  (d) Pollen grain slide.
5. Apply any immersion media appropriate for the objective lens and position the lens at roughly its focal distance away from the sample.
6. Begin live scanning and focus on the sample.
  **▲ CRITICAL STEP** If using a slide with features (for example, pollen grains), use a higher magnification so that the smallest feature chosen occupies ~50 × 50 pixels. If sized like this, it ensures that any small motion or pixel alignment errors will not corrupt the measurements. However, if magnification or laser power are too high, bleaching and damage to the feature may occur.
7. Open a live histogram of the pixel intensity values from the image.
8. Set the vertical scale (number of pixels) to ‘logarithmic’ to see the low pixel counts such as those between 1 and 100.
9. Set the *x* axis to show the full range of possible pixel values and adjust laser power and/or PMT settings so that live pixel values occupy 25% of the full intensity range. Once these laser power and PMT settings are set, they should not be changed for the duration of the measurements. If they change, the steps below will need to be repeated.
  **▲ CRITICAL STEP** High laser powers result in more photobleaching, which interferes with the interpretation of the measurement. The preference would be to first turn down the laser power, rather than the PMT voltage.
10. Adjust the pixel intensity range on the histogram to show 50% of the full range (Fig. 9), and record/remember the maximum pixel value. If the histogram display also shows the mean and maximum values of the image, record that too.
11. Continue imaging for at least 2 min and compare the current pixel values with the values noted in Step 10. If the values are considerably different, it shows that either the sample is bleaching and/or the system is not stable. Find either a more stable sample or turn the laser power down to decrease photobleaching, such that the pixel histogram is stable for ~2 min. ▲ **CRITICAL STEP** The signal needs to be stable to continue this protocol because the absolute pixel values need to be compared across time, as the dispersion compensation is adjusted.
12. Record the current dispersion compensation setting of the laser (usually in fs^2^ group delay dispersion) or external module and record the shape of the histogram alongside the mean and maximum pixel values (Box 5).
13. Change the dispersion compensation by a fixed amount (increments of ~2,000 fs^2^ GDD are generally sufficient).
14. Examine the live histogram and, again, record the shape of the histogram alongside the mean and maximum pixel values.
15. Continue repeating Steps 13 and 14 in the positive direction from the initial setting, and then repeat in the negative direction. If the mean pixel value drops >30% from the initial recorded value, do not continue in that direction.
**◆ TROUBLESHOOTING**
16. Plot the mean pixel values of the image as a function of dispersion compensation values (Fig. 10). Identify the GDD value that gives the highest mean pixel value.
17. Now repeat Steps 12–15 around the GDD value in Step 16 to find the exact best GDD compensation, this time with increments of~250 fs^2^ GDD. The dispersion compensation setting that gives the highest mean pixel value is the one you want to use for your experiments.
18. Repeat Steps 6–17 for the different wavelengths and objectives that will be used for imaging.
**◆ TROUBLESHOOTING**

### Procedure 6: measuring photomulitiplier tube performance

**• TIMING 3.5 h**

**Fig. 11:**
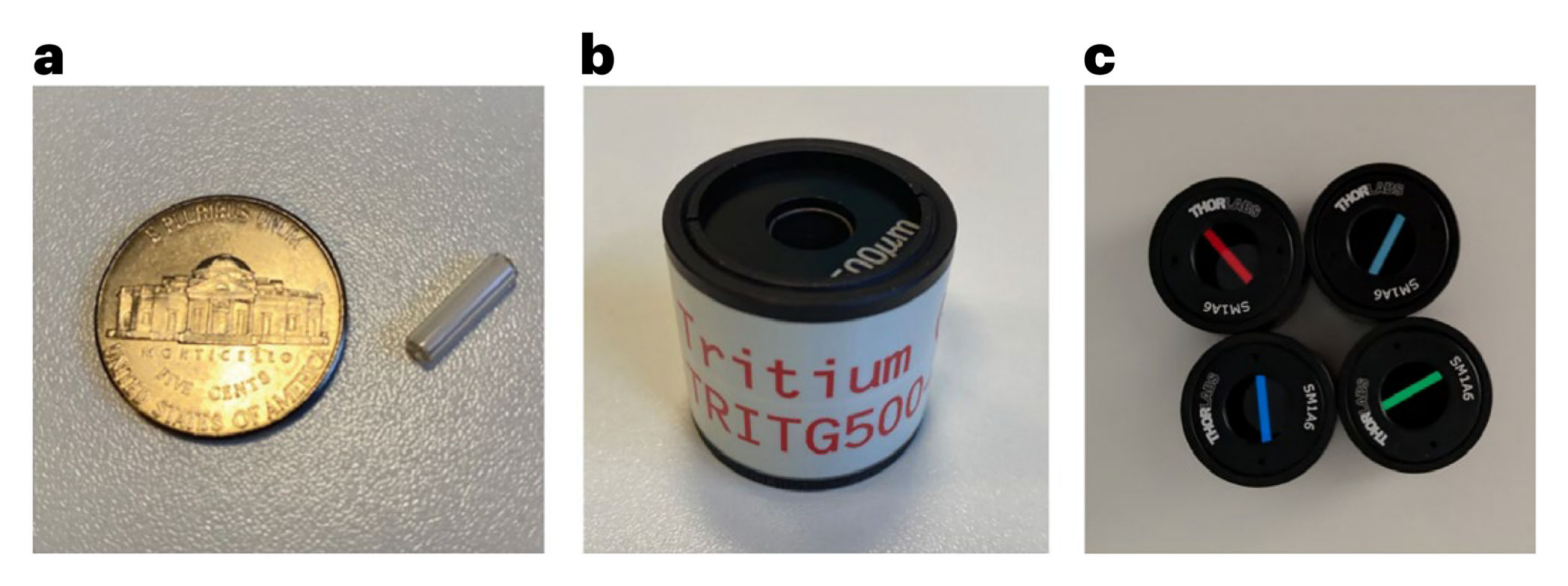
A hand-made tritium light source. **a**, A 3 mm × 11 mm tritium vial next to a 5 cent coin. **b**, The assembled tritium light source. The pinhole is at the top and will be placed immediately beneath the objective to test the entire collection system or PMT window to directly test the PMT. **c**, The multicolor assortment of tritium capsules, each with a different color phosphor (shown without a pinhole for clarity). SM1A6 Thorlabs parts were used here for a specific setup, which is not part of this procedure.

**Fig. 12:**
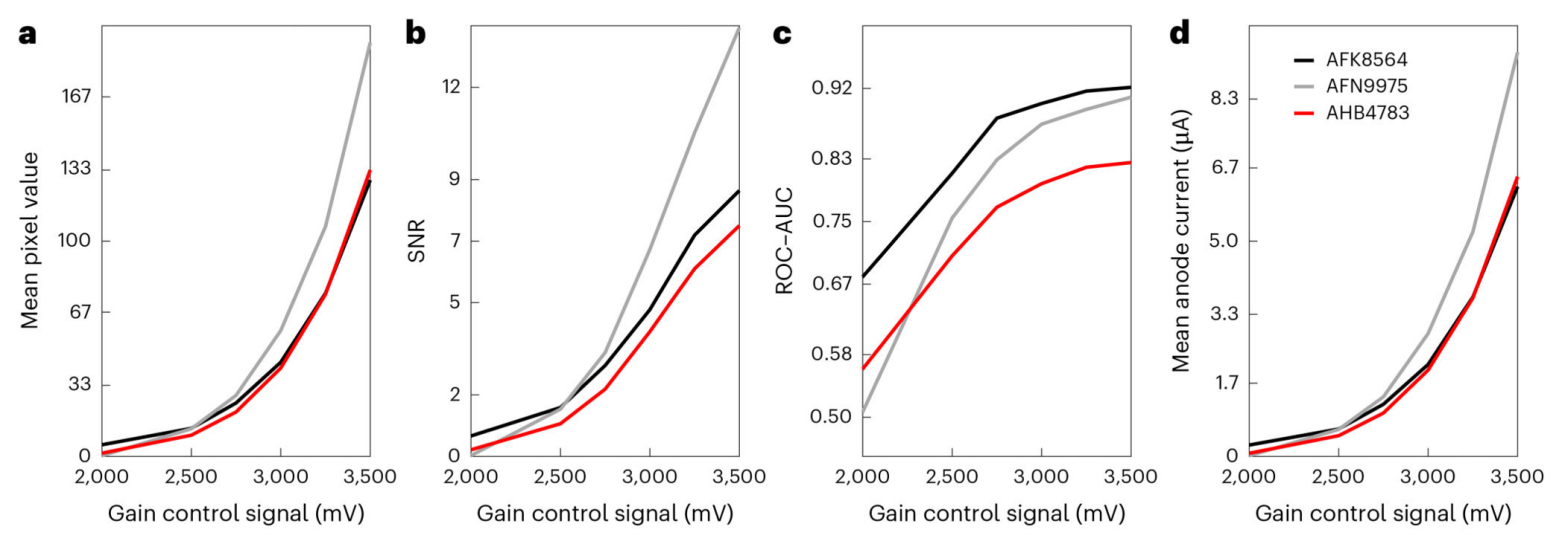
First-day performance for three multialkali PMT units of the same model. **a**–**d**, Mean pixel value (**a**), SNR (**b**), ROC–AUC (**c**) and mean anode current (**d**) data shown for three Hamamatsu R10699 PMTs on the first day of installation. PMTs were tested within the full collection optics system (green channel) of the same microscope. Gain setting is represented by the control signal voltage.

**Fig. 13:**
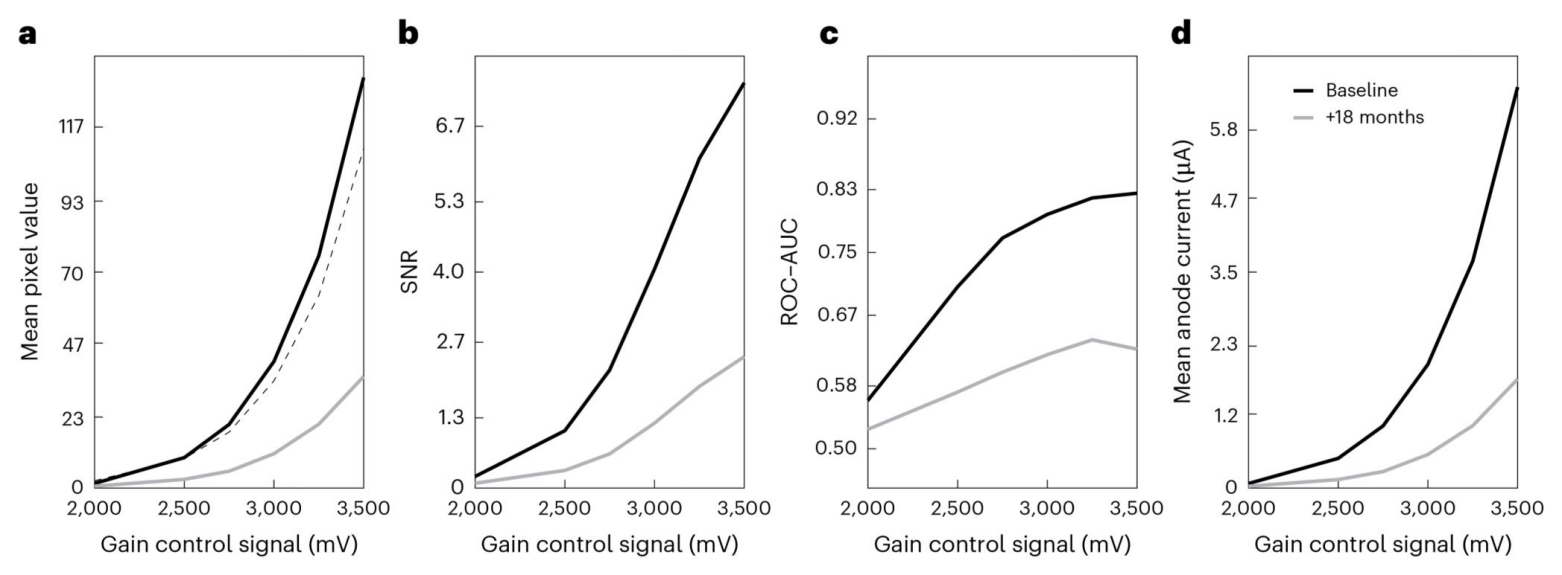
Change in PMT performance over time. **a**–**d**, Mean pixel value (**a**), SNR (**b**), ROC–AUC (**c**) and mean anode current (**d**) collected for a Hamamatsu R10699 PMT unit when it was first installed and after 1.5 years of routine use. The dashed line shows pixel values expected based upon tritium decay alone. The gain setting is represented by the control signal voltage.

**Fig. 14:**
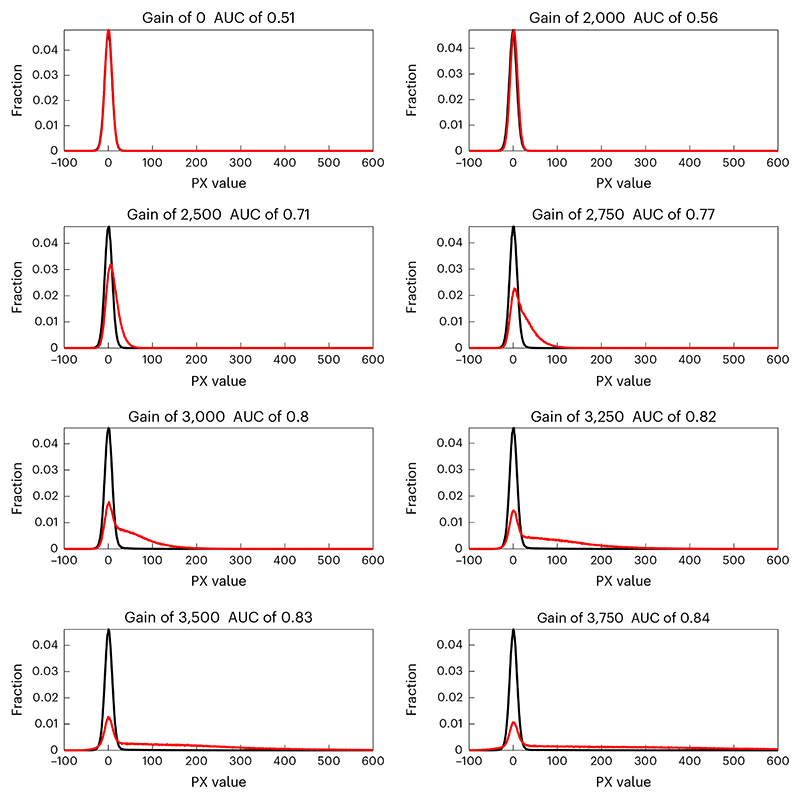
Pixel grayscale value distributions and ROC–AUC at a series of gain settings for an example multialkali PMT. For each gain setting, distributions of pixel (PX) values from dark response (black) and light response (red) images are shown. Corresponding ROC–AUC values are indicated at the top of each panel. The gain expressed as control signal voltage (mV).

**Fig. 15:**
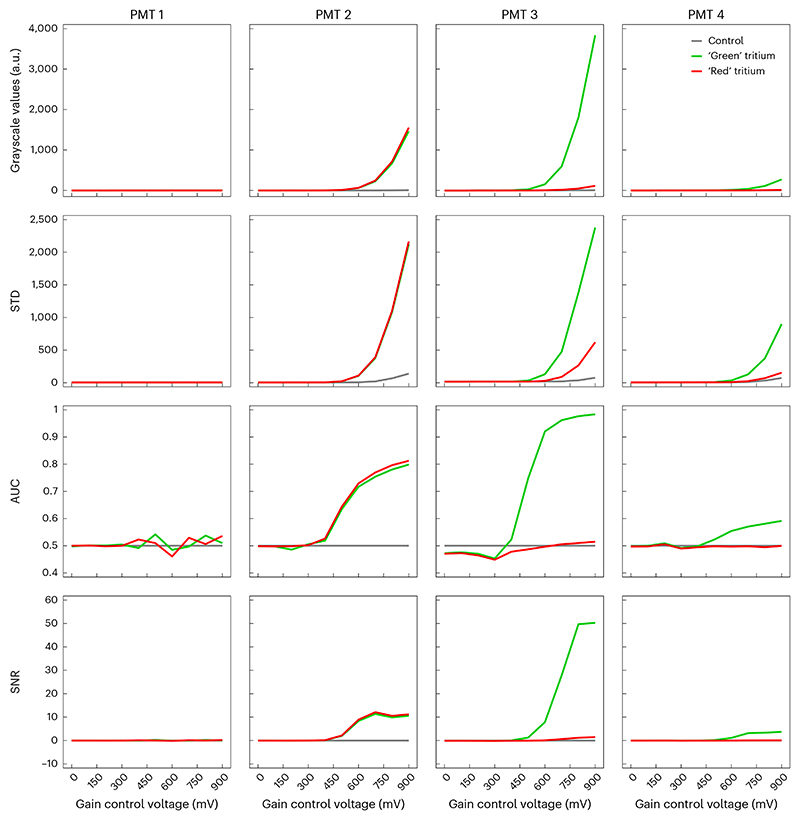
Comparison of two different GaAsP PMTs of the same model tritium light sources (red and green) and control (no light source). The PMTs **(Hamamatsu H10770PB-40)**. Mean (row 1), standard deviation (row 2), were measured with bandpass filters in place (PMT1: 570–620 nm bandpass; ROC–AUC (row 3) and SNR (row 4) for two example GaAsP PMTs for two different PMT2: 500–550 nm bandpass).

1. Prepare the tritium/phosphor light source:
  (a) Select a tritium vial of the appropriate color for the detection channel being tested (for example, ‘red’ or ‘green’) or use a ‘white’ tritium vial for testing multiple color channels.
  (b) Use a small drop of epoxy resin to affix the tritium vial to the center of the inside surface of the SM1 end cap and allow the resin to set overnight (Fig. 11).
  (c) Screw the end cap, with the mounted tritium vial, onto the lens tube.
  (d) (Optional) Add a neutral density filter to reduce light intensity and/or a diffuser to reduce inhomogeneity, if required.
  (e) (Optional) Add a narrow bandpass filter to limit the spectral bandwidth of the light source. This is recommended if the light source will be used to compare the collection system of different microscopes.
  (f) At the open end of the lens tube, attach a mounted pinhole (Fig. 11b).
  (g) Label the assembled tritium source with a unique reference number, assembly date and ‘color’ of the tritium vial (for example, ‘red’).
  (h) Once assembled, the tritium source can be used for many years. To ensure stability, it must not be assembled/disassembled and should be stored in a dust-free container.
2. Install the PMT in the microscope to test the entire collection system and install a microscope objective lens used for imaging experiments. Keep this objective lens consistent for all future readings (Box 6).
3. (Optional) Install the PMT in custom optomechanics at a fixed distance from the light source to test the PMT independently from the remainder of the collection system.
4. Turn on the microscope hardware and software necessary for controlling image acquisition.
  **▲ CRITICAL STEP** Refer to previous measurement metadata to ensure consistency of instrument software settings and hardware configuration across tests. Relevant factors include TIA settings, microscope hardware (including emission filters) and image acquisition settings.
  **▲ CRITICAL STEP** Take precautions to prevent any stray light from reaching the PMT by building a light-tight chamber to surround the microscope and switching off room lights. Additionally, switch off or close laser shutters to prevent any laser light from affecting measurements of PMT performance.
5. Operate the PMT at a normal gain (voltage) while shielding it from any light for around 1 h before testing to ensure stable operation.
  (Optional) For new PMTs or a PMT that has been unused for several months, it may be desirable to first ‘age’ the tube by operating it for several hours, as this can improve stability.
6. Make a record of the room temperature, because PMT performance is dependent on temperature.
  **▲ CRITICAL STEP** Room temperature should match that at which experiments are routinely conducted and be consistent within and across tests.
7. Measure the response of the detector under dark conditions (the ‘dark response’) by collecting a short time series of images (few seconds duration) at each PMT gain setting for a range of gain values. A time series is taken such that a middle-of-series single image can be used for analysis and avoidance of artefacts on the ‘start’ or ‘stop’ frame. For testing the Hamamatsu R10699 with C6270 socket assembly, a range of control voltages from 0 to 3.5 V in 0.25 V steps was used, which corresponds to 0–900 V across the PMT electrodes.
  **▲ CRITICAL STEP** An image must be acquired at zero gain to determine any image value offsets unrelated to the PMT itself.
  **▲ CRITICAL STEP** Care should also be taken not to exceed the maximum HV indicated on the datasheet for the specific model of PMT
8. Place the constant light source under the objective lens (if testing the entire collection system) or PMT window (for direct PMT tests).
  **▲ CRITICAL STEP** If using a tritium light source as described above, place the pinhole directly beneath the objective. Move the objective very close to the pinhole, for example, to the lip of the pinhole mounting. Do not use immersion media.
9. Stream live image data and slowly raise the PMT gain to a normal operating level (according to prior imaging experiments).
10. Monitor the grayscale values (mean or histogram) while adjusting the lateral position of the light source using the *X*/*Y* motorized stage controls to maximize the intensity of the image, thereby centering the pinhole on the objective lens.
  **▲ CRITICAL STEP** If this is a new tritium light source or a new model of PMT, take care to slowly increase the PMT gain. Image brightness (mean pixel grayscale value) should be similar to biological samples typically used in the laboratoy. If the images appear too bright, add a neutral density filter to the light source.
11. Collect a set of consecutive time-series images of the ‘light response’ at the same PMT gain settings used for the dark response tests in Step 7.
12. Calculate the ‘black point’ by computing the mean pixel value in the dark response image acquired at zero gain in Step 7.
13. Subtract the black point value from all the images of both the dark response and light response series.
14. Compute the mean and standard deviation across all pixels for each image.
15. Plot the mean pixel value at each gain setting (represented as either control signal voltage or HV) for the light response image series.
  **▲ CRITICAL STEP** It should be observed that the mean pixel value increases as a function of gain, owing to increased PMT amplification. In Fig. 12, which compares ‘day 1’ performance of three R10699 units, AFN9975 produces the highest pixel values as expected from it having the highest test sheet anode luminous sensitivity.
  **▲ CRITICAL STEP** Fig. 13 shows measurement data for a single unit (AHB4783 from Fig. 12) when first installed and then after ~18 months of routine use in one author’s laboratory. Mean pixel values have declined substantially over this time.
  **▲ CRITICAL STEP** If using a tritium light source, some decline in mean pixel value should be expected due to the radioactive decay of tritium. This can easily be computed by multiplying by a factor *k*, given by
  (3) $$k={\text{e}}^{-\frac{d}{\tau }},$$
  where *d* is the interval between measurements (in years), and *τ* is the decay time constant of tritium (17.75). The dashed curve (Fig. 13a) was obtained in this fashion and shows that the expected tritium decay would only account for a small fraction of the observed decline.
16. Compute signal-to-noise ratio (SNR) at each gain setting, *g*, as the difference in mean pixel value between the light response and dark response images, divided by the standard deviation of the dark response image as follows:
  (4) $${\text{SNR}}_{g}=\frac{\mu_{\text{light }}-\mu_{\text{dark }}}{\sigma_{\text{dark }}}.$$
  For example, Fig. 12 shows that SNR increases as a function of gain and that unit AFN9975 achieves higher SNR than the other two units. Figure 13 shows that SNR declines over time.
17. Perform a receiver operating characteristic area under the curve (ROC–AUC) analysis at each gain setting, using the distributions of pixel grayscale values in the light response image and corresponding dark response image. Figure 14 shows such pixel value distributions with the tritium source (red) and under dark conditions (black) at a variety of gain settings for an example PMT. At each gain setting, the ROC–AUC values (indicated at the top of each figure panel) quantify how well separated the two distributions are.
  **▲ CRITICAL STEP** ROC-AUC initially increases with gain before reaching a plateau (Fig. 14). Moreover, although unit AFN9975 has the highest anode luminous sensitivity, highest pixel values and highest SNR, it does not have the best performance as measured by ROC–AUC. Rather, unit AFK8564 has marginally better performance as judged by the fact it plateaus at a slightly higher ROC–AUC value. Unit AHB4783 is notably poorer, despite having similar pixel values and SNR versus the best performing unit, which is likely due to its high variance (not shown). Figure 13 shows that ROC–AUC values decrease substantially over 1.5 years of routine use. Figure 15 shows measurement data from two GaAsP PMT units (Hamamatsu H10770PB-40) installed in a microscope after multiple years of usage.

(Optional) The ROC–AUC analysis can also be used to guide the choice of PMT gain settings to use during imaging experiments. Specifically, the choice of gain setting should consider:

(a) ROC–AUC performance. Ideally a gain setting will be selected close to the plateau of the ROC–AUC curve.
(b) HV across the PMT electrodes must not exceed the maximum value stated on the datasheet and should ideally be 20% below this value. Excessive HV can cause field emission from the dynodes and substantially shorten PMT life^47^.
(c) Anode current should be kept within safe limits, typically no more than a few microamperes. Refer to the datasheet for specific PMT models.

Anode currents can be estimated at each gain setting using the mean pixel grayscale value of the light response image along with knowledge of the TIA and ADC settings by

(5) $$I_{\text{anode }}=\frac{\mu_{\text{pixel}}\times {\text{ADC}}_{\text{V}}}{{\text{ADC}}_{\text{px}}}\times {\text{TIA}}_{g},$$

where *µ*_pixel_ is the mean pixel grayscale value of the light response image at a given gain setting (*g*), ADC_V_ is the voltage that the digitizer will map to the highest grayscale value (for example, 1 V), ADC_px_ is the corresponding grayscale value (for example, 2,048) and TIA_*g*_ is the gain of the TIA (for example, 100 × 10^−6^A/V). Consider unit AHB4783 when it was first installed (Fig. 12, red curve). ROC–AUC increases with gain but starts to plateau at a control signal voltage of 3,250 mV. The corresponding HV is safely below the limit for this PMT model, and the mean anode current is acceptable at ~5 µA. Thus, this gain setting would be chosen for imaging experiments.

### Procedure 7: estimating absolute magnitudes of fluorescence signals

**• TIMING 3 h**

**Fig. 16:**
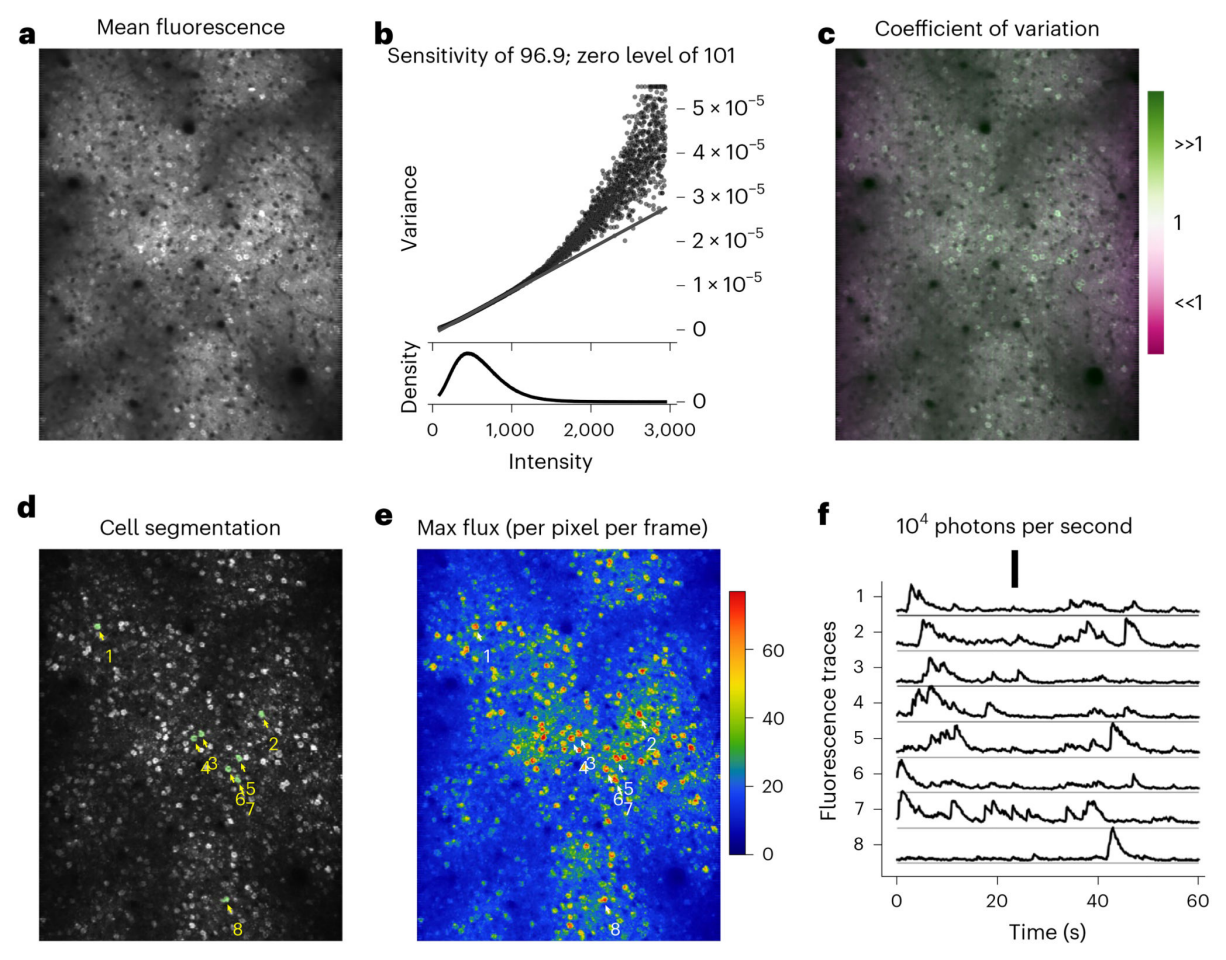
Photon transfer analysis. **a**, The average image of a 500-frame two-photon calcium imaging sequence in mouse visual cortex recorded at 8 frames per second. **b**, The PTC computed from the same sequence. It features a long linear portion corresponding to Poissonian noise dominating the frame-to-frame variance in all but the brightest regions. The slope of the robust linear fit (black line) reveals the photon sensitivity of 96.9 grayscale levels per photon. Note that the density of intensity values follows a long-tail distribution. The variance in bright regions of the image grows faster than predicted by the linear fit, reflecting the presence of physiological signals. The static images lack such deviations. **c**, The coefficient of variation (CoV) image reveals areas of higher variance than predicted from quantum noise alone. The calcium activity in cells produces a higher CoV, shading them green. **d**, Cell segmentation based on the maximum projection image; eight cells are delineated. **e**, The maximum photon flux per pixel expressed in the units of photons per pixel per frame. **f**, The fluorescence traces from the labeled cells expressed as photons per second. Scale bar, 10^4^ photons per second per cell.

**Fig. 17:**
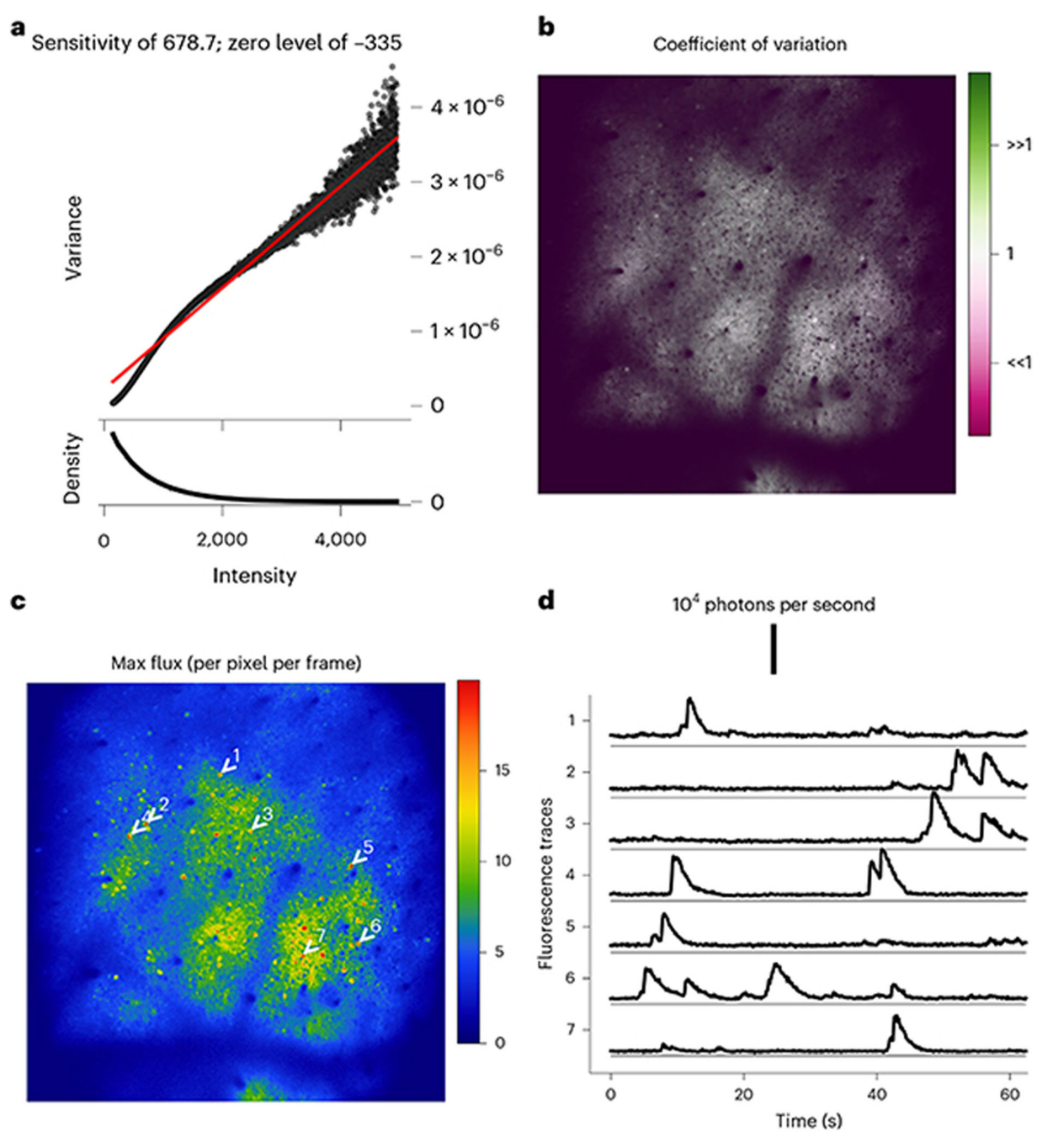
A different image sequence from another source. **a**, The PTC indicates that most pixels do not see a photon in each frame. The PTC has a non-Poissonian segment where no photons are detected. **b**, The coefficient of variation image reveals no deviations from predicted variance. **c**, The maximum photon flux is substantially lower than in our first dataset, due to the finer pixel pitch. However, the number of pixels per cell is about four times larger. **d**, After ROI averaging, fluorescence signals produced comparable peak amplitudes with the first data set.

1. Extract a sequence (*X*) of ~500 frames from a raw imaging sequence before any processing such as motion correction or filtering (Box 7).
2. (Optional) (applicable to systems with resonant scanners) Equalize the photon sensitivities by rescaling the image intensity according to estimated laser dwell times at each pixel to restore uniform photon sensitivity across the image. This step reverses the gain compensation performed by the acquisition system and can aid in more accurate photon sensitivity estimations. We did not include this step in our implementation example.
3. (Optional) Exclude regions near image boundaries where laser blanking and mirror vibrations might affect measurements. In our example implementation, we excluded four-pixel margins around image boundaries.
4. Determine the rounded mean values $M=\left[\frac{1}{2}X^{\prime }+\frac{1}{2}X\right]$ and the squared difference values $D=\frac{1}{2}{\left(X^{\prime }-X\right)}^{2}$, where *X’* is *X*delayed by one frame.
5. Construct count, intensity and variance vectors:
  (a) Let vector *I* represent all unique pixel intensity values in *M*.
  (b) Construct the count vector *C* so that each element of *C, C*_*j*_, contains the number of pixels *k* for which *M*_*k*_ = *I*_*j*_.
  (c) Compute the variance vector *V* so that each element of *V, V*_*j*_, contains the average value of *D*_*k*_ across all pixels *k*, for which *M*_*k*_ = *I*_*j*_.
    **▲ CRITICAL STEP** Calculating means and variances from the differences between adjacent frames offers a more precise method to isolate the uncorrelated quantum noise from influences such as neuronal activity. This approach is superior to alternative methods that estimate these values over extended time periods, as it better targets the specific characteristics of quantum noise.
6. Plot *V* against *I* to create the PTC (Box 8).
7. Determine the photon sensitivity *q* and the zero-intensity level *I*_0_ by performing a linear fit to the PTC so that *V ≈ q* x (*I* – *1*_0_). Ensure reliable results by weighing the fit with the pixel count vector *C* and employing a robust fitting method that minimizes the impact of outliers. Our procedure utilized the Huber linear regressor from the sklearn package in Python^76^.
**TROUBLESHOOTING**
8. Plot the linear fit alongside the PTC (Fig. 16b). Look for the characteristic linear portion where photon noise dominates. For static objects, the entire PTC should align with the linear fit. In dynamic experiments, expect a linear and tight component in darker regions and a quadratic, dispersed component in bright regions.
  **▲ CRITICAL STEP** The measured PTC (Fig. 16b) reveals a characteristic linear portion at the lower intensity range, indicating the Poissonian properties of the quantum noise. The upper intensity range is dominated by fluorescence signals such as neuronal activity and tissue motion, causing more dispersion with rapidly increasing variance. A robust linear fit (indicated by the black line) isolates the slope of the linear component, the photon sensitivity, with a value of 96.9, signifying that the system digitises images so that 96.9 grayscale levels are used to quantize the average intensity due to one detected photon. The intercept of the linear fit with the *x* axis designates the inferred true zero-intensity level.
9. For each pixel in the image, plot the coefficient of variation—the ratio of the pixel’s mean value to its variance.
  **▲ CRITICAL STEP** The coefficient of variation image serves as a diagnostic tool to detect diverse imaging anomalies such as pixel saturations and extra noise sources. Saturated or clipped regions, for instance, will exhibit low coefficients of variation whereas motion, laser fluctuations and physiological signals will produce high coefficients of variation in the bright regions of the image.
  **▲ CRITICAL STEP** Properly scaled by the photon sensitivity, the coefficient equals 1.0 for any Poisson process. In Fig. 16c, regions with exact Poisson noise prediction appear gray, higher variability regions appear green and lower-than-expected variance regions appear purple. For instance, green neuronal bodies reflect added variance from neuronal activity, and purplish bands along the frame’s edges result from the acquisition system’s compensation for slower laser scanning speeds near the boundaries. Since we estimate the average photon sensitivity across the entire image, the method overestimates the photon sensitivity close to edges where the system applies a lower gain. For the same brightness, more photons are detected and less noise results, producing a lower coefficient of variation. This compensation can be undone by using a more accurate local photon sensitivity estimation, although it was not performed here where we estimated the average photon sensitivity for the entire image for simplicity.
10. Rescale the original image sequence as $\hat{X}\leftarrow q\times \left(X-I_{0}\right)$ to create photon flux movies, where pixel values represent photon flux in units of photon counts per pixel per frame. Alternatively, rescale the images as $\dot{X}\leftarrow q\times \left(X-I_{0}\right)/(\text{d}x\text{d}y\text{d}t)$ for units of photons per square micron per second, where d*x* and d*y* denote the pixel pitch in microns, and d*t* denotes the frame period in seconds.
  **▲ CRITICAL STEP** Fig. 16d shows several segmented cells detected by thresholding a maximum projection image across time, subtracting the mean fluorescence. Figure 16e depicts the maximum projection image across the 500 frames expressed in units of photon flux. Note that the mean photon rates will be much lower. The density of intensities shown in Fig. 16b indicates that the majority of pixels have intensities on the order of 400, corresponding to (400 – zero level)/sensitivity = 3 photons per pixel per frame in this particular sequence.
11. (Optional) Correct the photon flux movie $\hat{X}$ for motion. The pixel interpolations performed by the motion correction algorithm will have a negligible effect on photon rate estimation. Other processing steps, such as temporal or spatial filtrations, might have more complex effects and require careful consideration (not covered here).
12. (Optional) For dynamic signals (for example, calcium imaging), extract the absolute fluorescence signal *y* by summing pixels over each region of interest *R* (for example, a cell) with equal weights:
(6) $$y=\frac{1}{\text{d}t}\underset{i\in R}{\sum \;}{\hat{X}}_{i}.$$
  The magnitude of trace *y* will be correctly expressed in units of photons per second.
13. (Optional) The signal*y* can also be computed using a weighted mask *h* as:
  (7) $$y=\frac{\gamma }{\text{d}t}\underset{i\in R}{\sum \;}h_{i}{\hat{X}}_{i}.$$
  Here, *γ* is the normalization coefficient for proper scaling of the photon sensitivities. This computation is not trivial for dynamic scenes. The optimal unbiased scaling coefficient is
  (8) $$\gamma =\frac{\sum_{i\in R}h_{i}{\bar{X}}_{i}}{\sum_{i\in R}h_{i}^{2}{\bar{X}}_{i}},$$
  where ${\bar{X}}_{i}$ is the time-averaged pixel value in $\hat{X_{i}}$.
  A simpler normalization $\gamma =\frac{\sum_{i\in R}h_{i}}{\sum_{i\in R}h_{i}^{2}}$ provides an accurate estimation when the image under the mask is approximately uniform. When the image is nonuniform, then this normalization results in a lowered estimation. We recommend using this simpler normalization.
  **▲ CRITICAL STEP** Measuring fluorescence in absolute units becomes valuable for monitoring signal quality across various experiments and laboratories. Figure 16f shows the photon rates for the regions of interest (ROIs) corresponding to the detected cells from Fig. 16d. Each ROI contains 18–24 pixels with uniform weights.
  **▲ CRITICAL STEP** It is important to ensure that no spatial or temporal filtration is applied to the images before the photon rate estimation since it can bias the estimation.
14. Repeat the method on a different system for direct comparison or on the same system at a later date to track performance.
  **▲ CRITICAL STEP** We applied the same method to a completely different dataset from another laboratory (Fig. 17). This sequence uses a different fluorescent dye, laser setting, optics and acquisition parameters. Here, the system applies higher gains to compensate for lower pixel dwell times, producing a photon sensitivity of 678.7 grayscale levels per photon (Fig. 17a). While the imaging setups differ substantially, we arrive at comparable magnitudes of the fluorescence signals with peak amplitudes reaching more than 104 photons per second from each cell (Fig. 17d).
**◆ TROUBLESHOOTING**

### Troubleshooting

Troubleshooting information can be found in Table 1

### Timing

Procedure 1, measuring laser power at the sample: 2 h

Procedure 2, FOV size: 2 h

Procedure 3, FOV homogeneity: 2 h

Procedure 4, spatial resolution: 3.5 h

Procedure 5, group delay dispersion optimization: 1.5 h

Procedure 6, photomultiplier tube performance: 3.5 h

Procedure 7, estimating absolute magnitudes of fluorescence signals: 3 h

### Anticipated results

These procedures should be carried out at regular intervals, such as once a month or every 3 months. All procedures, except procedure 1, should be repeated after changes are made to laser beam alignment. After installation, alignment can drift due to mirrors settling in place on an optical table. Therefore, procedures should be carried out much more frequently toward the start of the microscope system life to ensure a stable baseline is acquired. Frequency will depend on how often the microscope is altered or used; the below recommendations are based on a laboratory using their system daily for multiple hours. Procedure 1 is expected to change over time due to laser degradation, so should be performed once a month or preferably before every major experiment. Procedure 2 should be carried out once every 6 months to ensure nothing drastic has changed with the system, as it is unlikely that the fundamental properties of the image scale should change on their own. Procedures 3 and 4 should be carried out once every 3 months to ensure consistency. Procedure 5 needs only to be carried out once every 6 months. Procedure 6 should be carried out monthly to monitor PMT health. Procedure 7 should be carried out once every 6 months and is not essential to monitor performance but is a good indicator of performance changes.

## Figures and Tables

**Fig. 18:**
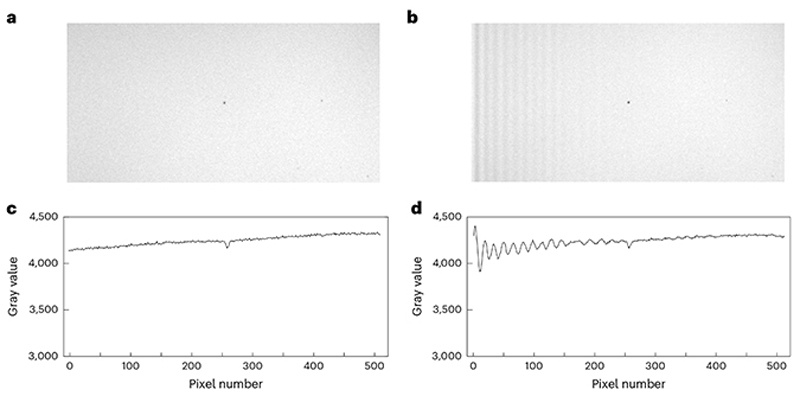
Pockels cell resonance effect. **a**, The image of a homogeneous fluorescent medium with no ringing effect. **b**, Same image as **a** but with Pockels cell ringing visible on the left side. **c**, A line profile along the yellow line in **a** that only shows a drop at the dark spot along the line. **d**, A line profile along yellow line in **c** that shows intensity oscillations on the left side of the image where Pockels cell ringing is present. This effect does not change with the zoom factor. The line profile is chosen to extend over a darker spot to highlight the magnitude of the ringing.

**Fig. 19:**
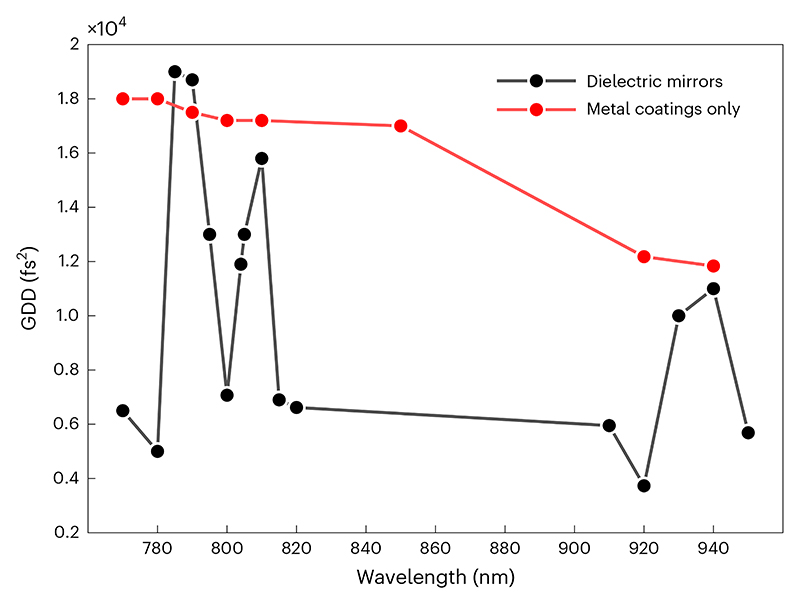
The effect of dielectric coatings on GDD. Optimal GDD was estimated using a fluorescent slide and the built-in GDD compensation on a Coherent Chameleon Vision II laser. The compensation curve was first measured with three dielectric coated mirrors (ThorLabs EO3) in the path (black line). Obvious sharp peakiness is seen in the required compensation value as a function of wavelength. After these three mirrors were swapped with metallic coated mirrors, the peakiness completely disappears (red line). The three EO3 mirrors did not contribute equally to the above effect (data not shown).

**Table 1:** Troubleshooting table

Procedure	Step	Problem	Possible reason	Possible solution
1	4	Laser power value changes over the course of the day by >5%	If the laser itself is stable, such a change could be due to instability of the power modulator. Pockels cells are temperature sensitive, and a unit with an internal or directly affixed beam dump can drift if the dumped beam causes strong local heating, especially for lasers with power outputs of a few watts	In this case, the beam dump should be decoupled from the Pockels cell, with a short distance between them. Caution: beam dump repositioning should only be attempted by a competent, trained, individual. The discarded beam exiting a Pockels cellis of high power and often exits at a dangerous angle, making an eye strike possible
1				Similar temperature instabilities can occur if the Pockels cell is downstream of a shutter and only gets the laser beam when imaging is active, as its internal temperature will change as the laser passes through the cell. Open shutters 30-60 min before measuring laser power
1	16	Laser power value changes over the course of minutes and is not stable	Improper use of thermal sensor heads	Thermal power sensors are sensitive to ambient temperature and physical handling, especially at lower power values. The sensor should be allowed to equilibrate and only used once zero drift has ceased. For laser powers under a few milliwatts, users should consider photodiode sensors, as they are less sensitive to external conditions and shielding ambient light is very straightforward, as compared with heat sources
1	22	Laser power has changed by more than 5% since the first recorded measurement	Laser throughput is changing	Measure laser power directly after the laser enclosure and compare to manufacturer’s commissioning values. If the laser power at the laser enclosure has changed by >5%, contact the manufacturer for assistance
1			Laser alignment has changed. Laser power at the objective is highly sensitive to beam alignment. For instance, if the beam is not hitting the scanners correctly, it can be partially clipped, reducing the laser power reaching the sample	Make a record of the laser power at different points in the beam path to determine if power is being lost. Make logs of these values at the different points along the path after each alignment change to make troubleshooting easier. Useful locations to log transmission would be before and after the modulation device as well as before and after the scan head
				Having alignment targets in two or more places on the table and one at the objective mount is also a simple way to troubleshoot if measurements are off due to alignment. If the beam is not hitting the center of a target, it suggests the laser is misaligned before that point. Adjustable aperture irises are convenient for this purpose, as they can be mounted directly in the path, yet opened for free beam passage under normal conditions. Make a photographed log of laser alignment at apertures and targets to correlate any changes in alignment with changes in laser power
1			The power sensor has not been calibrated recently	Power sensors may need calibration every 2–3 years; if your values are slowly changing, you may want to recalibrate your power sensor
2	13	Pincushion, barrel distortion and *z*-plane (field) curvature	Pincushion and barrel distortion are usually caused by optical aberration at high scan angles in excitation optics	Ideally, the *XY* beam displacement in the imaging plane depends linearly on the scan angle Practically, due to optical design compromises, this linearity breaks down at higher scan angles, usually toward the corners of the image Unfortunately, this is a limitation of the optics and is most easily resolved post hoc by correcting the images. For applications such as functional in vivo imaging it may not be necessary to correct for distortion
3	10	Unusually shaped inhomogeneity that extends along the microscope’s fast axis and does not change shape with zoom	One potential source of image inhomogeneity that is not related to the optical properties of the microscope is intensity ‘ringing’ that originates in a Pockels cell (Fig. 18). Some microscopes use an electro-optical power modulator known as a Pockels cell to rapidly control laser power through the application of large voltages to the Pockels cell. The materials used in these cells can have a strong piezo-mechanical effect, resulting in a damped oscillation at the cell’s resonance frequency. This is typically seen on the fast scan axis, on Pockels cells that do not have any additional damping (the so-called BK ‘clamped’ option on one common brand, Conoptics)	Disabling beam blanking should reduce or eliminate the problem if it originates from the Pockels cell. It is for this reason that we recommend initial vignetting measurements are done over a range of zooms. Note that Pockels cell resonance impacts on inhomogeneity may look different to that shown, as they will depend upon the specifics of how the sample is imaged and the nominal scan line duration
3	14/15	Local inhomogeneities	Bubbles in the immersion media or inconsistent fluorescence in the imaging sample	If using a slide, try cleaning it
3	14/15		Dirt on optics somewhere in the path, it is not unheard of for dust to burn on to optics due to high laser powers	Try using compressed air to dust the optics in the path to identify if any are causing issues or where possible directly observe lenses to check for burnt on dust
3	14/15	Asymmetric dropoff of the fluorescence toward the edges, or off-center peak	Problems with alignment	This can be confirmed with the measurement of spatial resolution (see Procedure 4). The geometry of the excitation volume will most probably be less tight in the area of the FOV that appears darker
3	14/15	Vignetting	Problems with pupil conjugation in the system	Evaluate whether the beam ’walks’ at the back pupil
4	5	Beads move around	Evaporation from the sample being left out too long	Seal the coverslip to the slide if planning to use the slide for two hours or more, or if movement of beads is seen
4	5	Beads appear damaged, bleach quickly or move around	Laser power is too high	The use of high power to locate beads may indicate inefficient multiphoton excitation. For efficient two-photon excitation, a transform-limited pulse in the imaging plane should be used, and thus it is suggested to optimize GDD compensation before this protocol (see Procedure 5)
4	7	Beads appear damaged, bleach quickly or move around	An overly zoomed-in FOV may result in photobleaching and in extreme cases, the excess heat deposited locally could melt the agarose	Zoom out further while maintaining oversampling (beyond the Nyquist limit) to ensure that the pixel size is still small enough to permit precise curve fitting. If this is not possible, turn the laser power down
4	10	Data do not fit a Gaussian curve due to a plateaued peak	The bead stack has too many saturated pixels	Operate below the saturation regime to achieve a proper fit and correct estimation of resolution
4	10	Resulting PSFs are tilted	Optical aberrations tend to increase away from the center of the FOV	One could use a built-in rotation function, such as ImageJ (’Rotate...’) and MATLAB (’imrotate’), to correct the tilt in the Gaussian fit
4	10	Curve fitting is poor	SNR is too low for the bead stack	At each *z*-position in the stack, several frames can be acquired and averaged to yield a higher signal-to-noise image
5	15	Signal intensity never declines in one direction, but instead, continues to rise until the limit of your dispersion compensation correction is reached	Not enough dispersion compensation in the system	Decide to accept the limit GDD value as the ’best’ correction or consider adding more dispersion through an additional external unit. If the user typically uses a fixed wavelength, multiple bounces between chirped mirrors provides a straightforward way to add dispersion^87^
5	17/18	Fluorescence intensity measurements display sharp peakiness across imaging wavelengths used in experiments (Fig. 19)	Presence of dielectric mirrors distorting the pulse shape. Complex coatings of dielectric mirrors can have very strong and very specific wavelength dependence of dispersion	Despite the high reflection efficiencies of multilayer coatings, the authors recommend using metallic mirrors if possible, or dielectric mirrors that are designed and calibrated for known amounts of dispersion. The total effect of dielectric coatings (particularly old ones) on pulse shape cannot be assessed without sophisticated instrumentation, replacing dielectric mirrors with metal-coated alternatives is better than simply ’correcting’ for GDD peakiness with a prechirper^88^
7	7	Nonlinear PTC	Extremely low photon rates can cause nonlinear PTCs. Our method can produce a nonlinear kink for rates near single photons per frame, complicating sensitivity estimation in dim images	Increase laser power, dwell time (lower scan speed) or fluorophore expression
7	7		Variances growing quadratically with intensity may dominate the PTC in cases of strong fluorescence signals	Repeat the procedure with a static fluorescent object if needed. However, in multiphoton imaging, we have not encountered such issues even after reanalyzing diverse datasets from multiple laboratories
7	7		Non-Poissonian noise sources, such as excessive electronic noise or laser fluctuations, can also result in a nonlinear PTC	These issues should be separately diagnosed and addressed to restore the imaging system to a photon noise-limited state
7	14	Low photon rates	On occasions, low photon rates, especially in somatic calcium signals, might be expected, particularly in deeper tissue imaging or large fields of view	Improvements can be achieved by multiple approaches: the incorporation of brighter fluorescent indicators, longer dwell times, increased laser power, optimization of excitation wavelengths, replacement of aged PMTs or improved beam alignment and the GDD parameters

## BOX 1 Time-averaged laser power measurement considerations

This procedure describes the measurement of time-averaged laser power at the sample, that is, after the objective lens. However, the average power is not the complete picture. The lasers used in multiphoton microscopy are pulsed, which enable vastly improved efficiency for multiphoton excitation^21,77^. The power during a pulse is known as the peak power, which is often orders of magnitude higher than the average power. The peak power can be easily estimated from the average power under the simplifying approximation that the pulse envelope (the ‘shape’ of the intensity profile of the laser pulse) is rectangular

(9) $$\;\text{Peak power}\;(\text{W})=\frac{\;\text{Average power}\;(\text{W})}{\;\text{Pulse duration}\;(\text{s})\times \;\text{Pulse repetition rate}\;(\text{Hz})}.$$

You should consider the peak power and pulse energy as well as average powers when comparing experiments on different microscopes and lasers. Two systems with the same average power could have vastly different peak powers, leading to different amounts of photobleaching, emitted fluorescence and photodamage. Similarly, with changes in laser repetition rate, the pulse energy can be very different for the same average power. The use of lower repetition rate lasers with high pulse energies is common for photostimulation and for three-photon imaging, where high peak powers are also often required.

The aim of this procedure is to equip anybody with a simple metric for monitoring the laser power reaching the sample on their multiphoton microscope, the time-averaged power. This protocol cannot be used to measure actual peak power, since this requires knowledge of the pulse duration (Procedure 5) and pulse envelope, which is a much more complicated measure.

The way power readings will be taken will depend on your hardware and software. There are three possibilities:

- Manual readings: the user must set the power level in software and record the laser power value reported by the power meter console. We have chosen to describe this method
- Software calibration: a calibration process provided by the microscope acquisition software. Note that the calibrated power in microscope software may not be accurate for low values. If you need low values, such as 5 mW, then you should check the correct percent power manually. Calibration curves are wavelength specific because laser maximum power and Pockels cell bias voltage both vary with wavelength.
- Custom software: a custom software tool that reads values from the power meter console while controlling laser power, that is, a Python script

## BOX 2 FOV size calibration sample

The sample can be a microscopy grid overlaid over a Chroma fluorescent slide or a copper grid used in electron microscopy. Copper grids are cheap, will emit light when stimulated with a multiphoton laser and are robust to damage. They can also be overlaid on top of a Chroma slide to create a negative image.

We note that there are commercial slides available, such as the Axiom Optics Argolight slides, which could also be used for such measurements. These types of slides have been used with good success for microscopes employing single photon excitation. They are far more expensive than the options we provide here and can be damaged by very intense laser pulses, so should be used with care.

## BOX 3 FOV size considerations

The maximum FOV of an imaging system is defined by the maximum scan angles in the *X* and *Y* dimensions and the excitation optics, but the working FOV used in experiments should be driven by the needs of the experiments and are often based on other characteristics, such as required sampling density, field homogeneity (Procedure 3), field curvature and distortion. The FOV size is also used in mosaic imaging where tiles of images are acquired in a grid and merged. FOV size is not expected to change from year to year, so monitoring its size is not required unless the optics or scanners in the excitation path of the microscope have been changed. Here, we describe how to measure the maximum effective FOV of a laser scanning multiphoton microscope.

We will measure physical dimensions of the imaged FOV at the minimum zoom factor (the largest possible scan angle) and evaluate field distortion based on that image. For this, we will acquire an image of a fluorescent sample with features of known geometrical dimensions and convert this to microns in the *X* and *Y* axes.

## BOX 4 Spatial resolution considerations

A standard method to measure the excitation volume shape is to image fluorescent beads that are small enough to approximate an ‘impulse’ function. That is, fluorescent beads that are smaller than the expected excitation volume. Beads with a diameter of 0.2 µm are commonly available and are smaller than most multiphoton systems’ excitation volume when near-IR (>700 nm) light is used. These are sometimes called ‘subresolution’ beads. The beads can be spread across a microscope slide or embedded in a three-dimensional tissue phantom^78^ (for example, agarose). Of course, even these small beads are not true impulse functions, but in practice, they are a close enough approximation that the resulting measurements are useful for characterizing a system. Imaging small structures in vivo can provide a rough proxy for bead measurements. However, biological features are not proper impulse functions either and they are typically extended in some dimension. Moreover, a nonzero background signal can distort excitation volume measurements from small structures in vivo. Therefore, measurement using subresolution fluorescent beads on a slide provides a better baseline for comparisons over different timepoints and different imaging systems.

Coverglass thickness is an important variable in measuring spatial resolution due to spherical or chromatic aberration increasing with coverglass thickness. This is especially true with higher NA objective lenses and those designed for air or water immersion, for example. The coverglass thickness should remain the same for comparisons over different timepoints and different imaging systems.

## BOX 5 Group delay dispersion considerations

The art and practice of dispersion control is rich and strongly dependent on the equipment at hand^79–81^. Beyond that, there is a plethora of instantiations for building external compensation modules, or general dispersion control, the details of each is beyond the scope of this protocol, especially if higher-order dispersion control is necessary.

This procedure will assume that the user has a form of an easily tuneable dispersion control module and will use the level of fluorescence generated following two-photon excitation as a surrogate for measuring changes in the pulse width. The goal is to collect image intensity data throughout the range of group delay dispersion (GDD) correction values and, ideally, find the dispersion compensation setting that corresponds to the brightest image. This would correspond to the shortest laser pulse that your system can deliver and, hence, the highest peak intensity of the laser for a given average laser power. The fluorescence can be used because the signal is proportional to the laser intensity squared, while the intensity is proportional to the average power/pulse width, as described earlier. If the laboratory has invested in an autocorrelator, using that will provide a more quantitative assessment of the pulse width, though the method described above is a reasonable alternative for fast optimization.

If you do not have either dispersion control nor an autocorrelator, how concerned should you be about laser pulse management? Dispersion management/compensation becomes less important for simpler optical paths and longer initial pulses (due to pulse broadening being nonlinearly proportional to the pulse width). If you have a relatively simple microscope, with few optical elements (for example, mirrors, galvanometer-based mirror scanners, scan lens, tube lens and objective) between your laser and the sample, and your laser has initial pulses of ~150 fs, dispersion compensation may not be a worthwhile investment. If neither of these conditions hold, it may be worthwhile to consider working with either another laboratory or a vendor to measure the laser pulse on sample with an autocorrelator to see if additional measures are needed. This is especially important for short pulse laser systems. For example, for a system with a total GDD of 6,000 fs^2^, a 60 fs pulsed laser will be broadened to 284 fs, an almost 5× increase and concomitant 5× decrease in total fluorescent generated. Whereas, a 150 fs laser pulse will be broadened to 186 fs, only a 1.24× increase in pulse duration and 1.24× less total fluorescence. We note that for intensity levels below saturation, though the instantaneous two photon efficiency drops by the square of the pulse duration increase, the total generated fluorescence loss scales linearly with pulse width through Eq. 1, as the relative duty cycle increases with increasing pulse duration.

## BOX 6 Considerations for measuring photomultiplier tube performance

In this procedure, we describe a simple protocol to quantify light detection performance. With minor variations, the procedure can be used to test either the entire collection system (defined here as objective, collection optics and PMT) or test a PMT directly. Direct tests of the PMT require it to be disconnected from the microscope but otherwise require minimal modification to the procedure. We note that this procedure does not provide measurements in physical units, nor does it test different aspects of PMT performance as might be reported on the datasheet (for example, anode luminous sensitivity), which requires additional sophisticated instrumentation and is beyond the means of most research laboratories. Instead, we present a procedure that provides a robust, relative measure of performance that can be used comparatively, for example, to compare several PMT units of the same model or monitor performance of the same unit over time.

The measure is based on ideas from signal detection theory and is not specific to PMTs; indeed, the protocol can be applied to any light detector (for example, hybrid detectors). Finally, it also allows the user to select the optimal operating point (typically referred to as ‘gain’) for a specific PMT unit. The gain of a PMT is adjusted by changing the HV (high voltage; typically 500–1,500 V) that is distributed across its electrodes. In most instances, the user will control HV via a lower voltage control signal (for example, 0–5 V) that is provided to the socket assembly or PMT module. Users should be aware of how gain is controlled on their specific system. We recommend performing this procedure at least once every 6 months for routine monitoring of PMT performance.

This protocol requires a light source with a stable output. Such sources are available commercially, for example, based on closed-loop control of an LED, or can easily be assembled using a tritium vial as an approximately constant light emitter^82^, as we have chosen to do for this procedure. Tritium is a radioactive isotope of hydrogen with a half-life of 12.3 years. Tritium vials contain a small amount of tritium gas and a phosphor coating that produces light by radioluminescence when bombarded with beta particles. Vials are widely available with phosphor coatings that produce various colors including ‘red’ and ‘green’. These work well over the duration of most experiments, although additional care must be taken when analyzing measurements made across years; first, the half-life of tritium will cause a well-defined decrease in intensity, and second, phosphor degradation will further impact long term stability^83^.

To evaluate the performance of a detector, we need to consider not only the measurements made with the light source but also the background response obtained under dark conditions. Dark currents increase with gain, vary between PMT units and can change over the lifetime of the PMT.

Although pixel grayscale values and SNR are informative, we recommend using ROC–AUC analysis as the best means to compare PMT units and track performance over time. Based on ideas from signal detection theory, ROC–AUC analysis enables comparison of the pixel value distributions obtained with the light source versus under dark conditions: a good detector will produce separated distributions with minimal overlap, which will be reflected by a large ROC–AUC value. SNR partially addresses this but does not consider the distribution of pixel values in light response images, which is a product of several factors including shot noise, multiplication noise and gain.

We illustrate the data analysis and interpretation using:

- Measurement data from several units of the same multialkali PMT model (Hamamatsu R10699) when first installed (‘day 1’ performance)
- Measurement data from a single R10699 when first installed and then after a long period of routine use
- Measurement data from two GaAsP PMT units (Hamamatsu H10770PB-40)

## BOX 7 Estimation of photon sensitivity

Light, with its stochastic, quantal nature, leads to unavoidable fluctuations in measured signal intensities, commonly termed as ‘shot noise,’ ‘quantum noise’ or ‘Poisson noise.’ An optimized microscope will function in a photon noise-limited mode, where other sources of noise have been minimized, making only the inevitable quantum noise relevant.

The statistical features of quantum noise follow the Poisson probability distribution, as photon detections are independent discrete events. However, additional factors such as the high-frequency pulsing of laser power, the PMT’s inherent gain stochasticity and noise from the amplification process add complexities. As a result, photons do not appear as discrete events in the signal, and for a given number of detected photons, the recorded pixel intensity will vary substantially. Additionally, the analog-to-digital converter (ADC) may introduce a bias and additional non-Poissonian electronic noise.

Yet, despite these complexities, the detected noise retains its essential Poissonian trait: the variance of quantum noise is linearly proportional to signal intensity, and the slope of this linearity reveals the photon sensitivity, the average increase in measured light per detected photon. Other sources of noise may add on top of quantum noise, but they may be recognized and isolated by their non-Poissonian traits. Finding a linear dependency between signal intensity and its variance provides a strong indication of the quantal nature of the noise. Accurately estimating the photon sensitivity allows effective translation of measured intensities into photon counts.

The following procedure estimates the photon sensitivity directly from experimental data. For more detailed and deliberate diagnostics and troubleshooting, the investigators are encouraged to use a standard static fluorescent preparation featuring a wide range of fluorescence levels, following the same procedure. The photon sensitivity is a stable property of the system since it is controlled by the PMT gain and gains applied by the acquisition system; it is insensitive to many other aspects of the imaging configuration such as magnification, the NA, laser power and so on. However, the photon transfer curve (PTC) and the photon counts can describe both the quality of the fluorophore expression and the microscope’s imaging performance.

## BOX 8 Photon transfer curve

The principal tool for photon sensitivity estimation is the PTC, which plots the variance of detected intensities against intensity values^84^. It is a remarkable fact that the relatively simple calculation of PTC provides so much insight into the properties of the image acquisition process in diverse imaging scenarios. The PTC can be derived from imaging a static fluorescent object over a short time and estimating the noise variance across a wide range of detected intensities.

However, for convenience, we can calculate the PTC during regular experiments in the presence of physiological signals and motion. In this case, the effects of the quantum noise must be isolated from other sources of signal variance. In contrast to quantum noise, dynamic fluorescence fluctuations, such as neuronal activity or tissue movement, produce temporal variances that scale quadratically with intensity. They also have correlated spatial and temporal structures, whereas quantum noise stays decorrelated. The ability to differentiate between the Poissonian quantal noise from other sources of variance enables robust estimation of photon sensitivity even during regular experiments in the presence of physiological signals and motion, without the need for a separate procedure. Estimating the PTC from the experimental data ensures consistency with acquisition settings, contributing to the efficiency of the process. For more deliberate troubleshooting, repeating the procedure with a static fluorescent preparation helps in producing more accurate results.

Figure 16 presents and Fig. 17 the outcomes of PTC estimation derived directly from experimental data using a 500-frame sequence taken from the MICRONS dataset^85,86^ and following the procedure described here. The implementation example can be accessed at the GitHub repository https://github.com/multiphoton-tools/compress-multiphoton. This implementation includes examples from a variety of projects, accessed through the DANDI Archive.

## Key points

- The best practices for the quantification of the performance of multiphoton microscopes are described, necessary to overcome the wide user- and facility-dependent variations in microscope calibration, which inevitably affect the reproducibility of data across laboratories.
- The procedures cover laser power, pulse width optimizations, field of view, resolution and photomultiplier tube performance and the majority should be carried out on a regular basis for maintenance of the system.
